# NLRP3 inflammasome suppression improves longevity and prevents cardiac aging in male mice

**DOI:** 10.1111/acel.13050

**Published:** 2019-10-18

**Authors:** Fabiola Marín‐Aguilar, Ana V. Lechuga‐Vieco, Elísabet Alcocer‐Gómez, Beatriz Castejón‐Vega, Javier Lucas, Carlos Garrido, Alejandro Peralta‐Garcia, Antonio J. Pérez‐Pulido, Alfonso Varela‐López, José L. Quiles, Bernhard Ryffel, Ignacio Flores, Pedro Bullón, Jesús Ruiz‐Cabello, Mario D. Cordero

**Affiliations:** ^1^ Research Laboratory Oral Medicine Department University of Sevilla Sevilla Spain; ^2^ Centro Nacional de Investigaciones Cardiovasculares Carlos III (CNIC) Madrid Spain; ^3^ CIBER de Enfermedades Respiratorias (CIBERES) Madrid Spain; ^4^ Departamento de Psicología Experimental Facultad de Psicología Universidad de Sevilla Seville Spain; ^5^ Centro Andaluz de Biología del Desarrollo (CABD) Universidad Pablo de Olavide‐CSIC‐Junta de Andalucía Sevilla Spain; ^6^ Institute of Nutrition and Food Technology "José Mataix Verdú" Department of Physiology Biomedical Research Center University of Granada Granada Spain; ^7^ Laboratory of Experimental and Molecular Immunology and Neurogenetics (INEM) UMR 7355 CNRS‐University of Orleans Orléans France; ^8^ IDM University of Cape Town Cape Town South Africa; ^9^ CIC biomaGUNE San Sebastian‐Donostia Spain; ^10^ IKERBASQUE Basque Foundation for Science Bilbao Spain; ^11^ Universidad Complutense Madrid Madrid Spain

**Keywords:** autophagy, cardiac aging, longevity, morbidity, mortality, NLRP3‐inflammasome

## Abstract

While NLRP3‐inflammasome has been implicated in cardiovascular diseases, its role in physiological cardiac aging is largely unknown. During aging, many alterations occur in the organism, which are associated with progressive impairment of metabolic pathways related to insulin resistance, autophagy dysfunction, and inflammation. Here, we investigated the molecular mechanisms through which NLRP3 inhibition may attenuate cardiac aging. Ablation of NLRP3‐inflammasome protected mice from age‐related increased insulin sensitivity, reduced IGF‐1 and leptin/adiponectin ratio levels, and reduced cardiac damage with protection of the prolongation of the age‐dependent PR interval, which is associated with atrial fibrillation by cardiovascular aging and reduced telomere shortening. Furthermore, old NLRP3 KO mice showed an inhibition of the PI3K/AKT/mTOR pathway and autophagy improvement, compared with old wild mice and preserved Nampt‐mediated NAD^+^ levels with increased SIRT1 protein expression. These findings suggest that suppression of NLRP3 prevented many age‐associated changes in the heart, preserved cardiac function of aged mice and increased lifespan.

## Graphical abstract

In this study, we have shown the potential role of the NLRP3 inhibition to modulate several anti‐aging mechanisms during cardiac aging. This work highlights the importance of examining the therapeutic and prophylatic value of small molecule inhibitors of NLRP3 and its effects as potential therapy for preventing cardiac and metabolic aging.

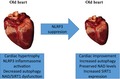

## 1 INTRODUCTION

Cardiovascular diseases (CVD) constitute the leading cause of death worldwide, with high prevalence in industrialized and low‐ to middle‐income countries (Ford, Li, Zhao, Pearson, & Capewell, 2009). Several risk factors have been identified, including genetics, hypertension, obesity, smoking, and physical inactivity. Secondary risk factors correlated with cardiovascular risk include insulin resistance and lipid profile (Gómez‐Pardo et al., 2016). However, aging, a natural process, poses the largest risk factor for cardiovascular disease (Fontana, 2008). During aging, we suffer a progressive impairment of several metabolic pathways that define body composition, insulin resistance, mitochondrial and autophagy dysfunction, and inflammation (Finkel, 2015). Many of these alterations are implicated in cardiac aging and age‐related cardiovascular diseases (North & Sinclair, 2012).

Markers of inflammation have been associated with cardiovascular diseases and proposed as other cardiovascular risk factors (Bullón et al., 2017). Recently, the role of the NLR family pyrin domain containing 3 protein (NLRP3) inflammasome has been studied in cardiovascular diseases. NLRP3 inflammasome is upregulated after myocardial infarction, atherosclerosis, ischemic heart disease, diabetic cardiomyopathy, chronic heart failure, and hypertension, and recently, NLRP3 and IL‐1β have also been proposed as new cardiovascular risk biomarkers (Bullón et al., 2017; Liu, Zeng, Li, Mehta, & Wang, 2017). Previous studies have suggested a role for NLRP3 inflammasome in several events associated with aging. Genetic deletion of NLRP3 in mice has been shown to improve healthspan by attenuation of multiple age‐related degenerative changes, such as glycemic control, bone loss, cognitive function, and motor performance (Youm et al., 2013). Furthermore, the deletion of NLRP3 in old mice increased muscle strength and endurance and prevented from age‐related increase in the number of myopathic fibers (McBride et al., 2017). However, the role of the NLRP3 inflammasome in lifespan and cardiac aging has not been studied. Hence, we sought to determine whether or not genetic deletion of NLRP3 may have effect on lifespan and potentially prevent cardiac aging.

## 2 RESULTS

### 2.1 NLRP3 deletion improves lifespan and metabolic aging

**Figure 1:**
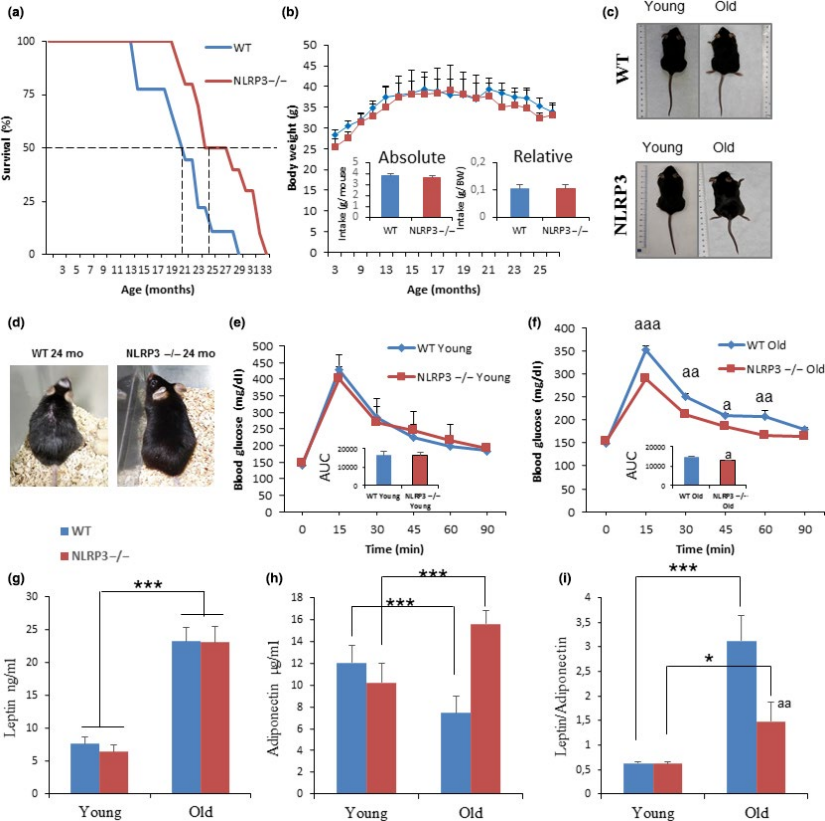
NLRP3 signaling suppression in mice extend lifespan and improve metabolic homeostasis. (a) Kaplan–Meier graph showing a significant increment of the máximum lifespan in WT mice (blue) compared with NLRP3 −/− mice (red). (b, c) Body weights and average daily oral food intake normalized to body weight and to mouse of the groups over time. Images of representative mice to illustrate phenotypic body mass of the groups at 20 months of age. (d) Representative photographs of 24 months of age mice. (e, f) Oral glucose tolerance test with area under the curve (inset). (g–i) Levels of leptin, adiponectin, and ratio in plasma. Blood samples were collected after overnight fasting. All data are presented as means ± *SEM*, *n* = 10 mice; *p < .05, **p < .005, ***p < .001 young vs. old mice. ^aa^ *p* < .005, WT vs. NLRP3 −/− mice

To evaluate the impact of NLRP3 deletion on survival and metabolic changes during aging, we followed NLRP3 deficient (NLRP3 −/−) and NLRP3 +/+ littermate control (WT) mice throughout the entire lifespan. The survival of NLRP3 −/− mice compared to littermate controls using a Kaplan–Meier survival curve was augmented with an increase in mean lifespan of 34% and in maximum lifespan of 29% (Figure 1a), while body weights and food intake did not differ between the two groups during the entire observation period (Figure 1b,c). Twenty‐four‐month‐old WT animals displayed increased age‐related alopecia than their coveal NLRP3 knockout mice (Figure 1d). Old NLRP3−/− mice exhibited a significant decrease in glucose at the OGTT peak (>15 min), compared with old WT mice (Figure 1e,f), indicating a higher glucose tolerance as measured as a trend toward lower values of the area under the curve (AUC) of the glucose tolerance test (insert of Figure 1f). Fasting blood glucose and circulating IGF‐1 levels were reduced in young and old NLRP3−/− mice, indicating that the insulin sensitivity of these animals was considerably higher than sham controls during aging (Table S1). Reduced levels of glucose and IGF‐1 have been associated with stress resistance and an antiaging effect (Brandhorst et al., 2015). Furthermore, leptin is an established regulator of body weight, and leptin/adiponectin dysregulation has been associated with cardiovascular disease, metabolic syndrome, and nonalcoholic fatty liver disease (DiNicolantonio, Lucan, & O'Keefe, 2016). Young and old NLRP3−/− mice showed similar serum levels of leptin compared to young and old WT, but a reduced leptin/adiponectin ratio with increased levels of adiponectin was observed in old NLRP3−/− mice (Figure 1g–i). Plasma lipid levels were reduced in NLRP3−/− old mice, accompanied by a significant reduction in hepatic transaminases, creatine phosphokinase, and lactate dehydrogenase (Table S1). However, plasma IL‐1β levels were not detected in old mice, but increased protein levels of active caspase 1 and IL‐1β were observed in old WT, when compared to NLRP3−/− mice (Figure S1), and increased levels of TNF‐α, IL‐6, and IL‐8 were observed in WT similar old mice and NLRP3−/− mice. This shows that the loss of NLRP3 did not affect the age‐related increase of other inflammatory pathways and confers an important role on inflammasome in cardiac aging (Table S1).

### 2.2 NLRP3 deletion preserved cardiac integrity

**Figure 2:**
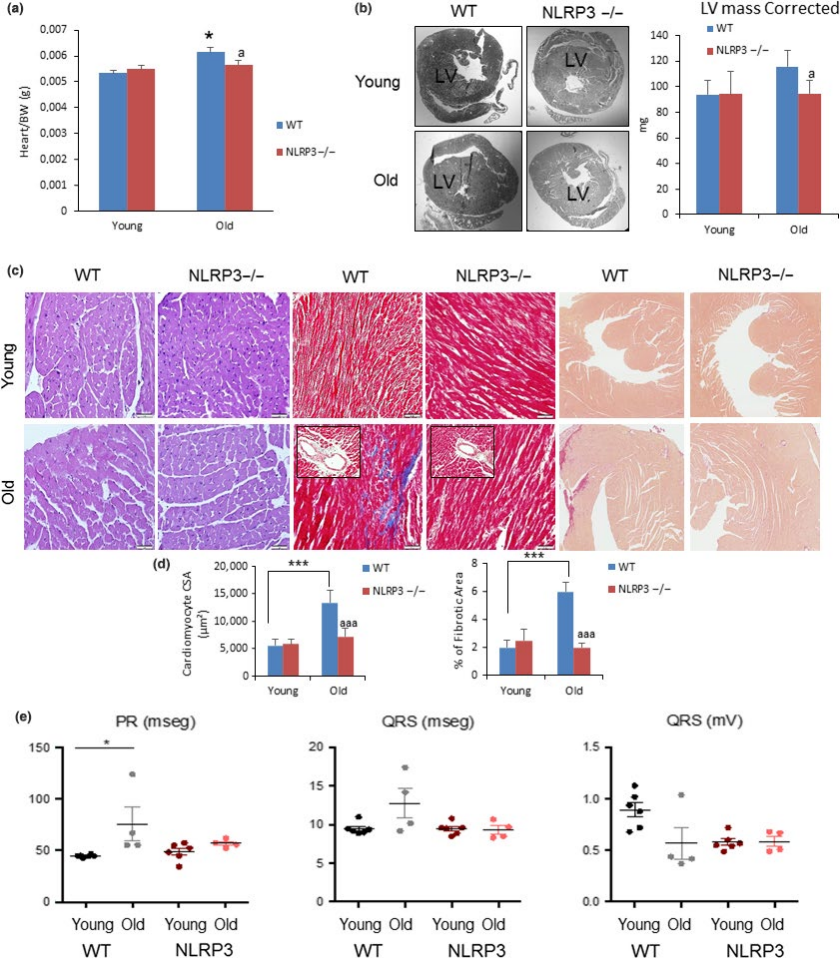
NLRP3 signaling suppression in mice induces cardiac protection. (a) Heart weight normalized to body weight. (b) Representative image of centripetal concentric LV hypertrophy (c) Representative Hematoxylin and eosin (H&E)‐stained section (left) of cardiac tissues from young and old WT and NLRP3 −/− mice. Representative Masson trichrome‐stained section (center) and Sirius red‐stained section (right). (d) Quantitative analysis of cardiomyocyte cross‐sectional (transverse) area with measurements of ≈100 cardiomyocytes and fibrotic areas from 3 to 6 mice per group. ***p < .001 young vs. old mice. ^aaa^ *p* < .001, WT vs. NLRP3 −/− mice. (e) Summary of differences in PR and QRS intervals in young and aged WT vs. NLRP3 −/− mice. All data are presented as means ± *SEM*, *n* = 10 mice; *p < .05

Heart weight normalized to body weight was increased in old mice in comparison with young mice, and heart weight was higher in WT in comparison with NLRP3−/− (*p* < .05) (Figure 2a and Table S2). Cardiac hypertrophy measured by the left ventricular wall thickness was significantly increased in elderly WT when compared to NLRP3−/− mice which was corroborated by LV mass measured by echocardiography (Figure 2b). To assess the impact of aging and cardiac hypertrophy on myocardial histology, the cardiomyocyte cross‐sectional area and fibrosis were quantified. In the hematoxylin‐and‐eosin–stained sections, aged WT mice showed an increased cardiomyocyte transverse cross‐sectional area unlike NLRP3−/− mice (Figure 2c,d), which was corroborated by wheat germ agglutinin staining (Figure S2). Further examination with Masson trichrome and Sirius red staining revealed overt interstitial and perivascular fibrosis in the aged WT group, with no significant changes in the aged NLRP3−/− group (Figure 2c,d). Ultrastructural analysis of the left ventricle in WT and NLRP3−/− showed mitochondrial abnormalities. We compared electron microscopic images of cardiac tissues from young and old WT mice and NLRP3−/− mice. TEM studies revealed evidence of mitochondrial damage in aged WT myocardium, including mitochondrial disarray, degeneration, fragmentation, reduction of mitochondrial area, and cristae disorganization, that is,. pointing in varying oblong and oblique directions in the matrix (Figures S3a,b and S4a,b).

Upon electrocardiographic examination, the mean QRS complex was not significantly wider in old WT mice; however, we observed a significant prolongation of the age‐dependent PR interval, which is associated with atrial fibrillation by cardiovascular aging (Figure 2e) (Magnani et al., 2013). Due to the improvement in the cardiac function of old KO mice and the low incidence of cancer in these mice (WT showed an increased rate of hepatocarcinoma and adenocarcinoma at death), the cause of death was unknown and will require further study.

### 2.3 Age‐associated metabolic changes were prevented in NLRP3−/−

**Figure 3:**
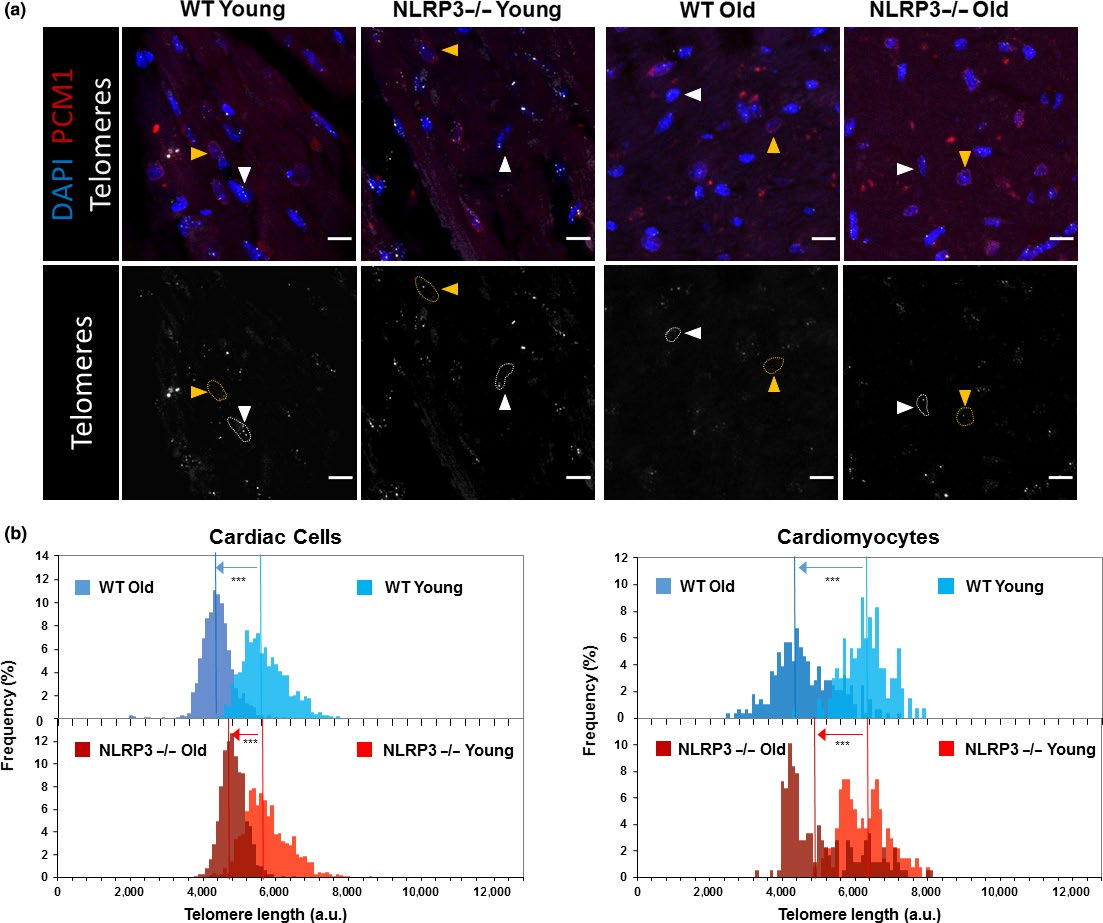
Telomere length diferentes in cardiac cells from WT and NLRP3 −/− mice. (a) Representative confocal images of telomere Q‐FISH and PCM1 immunofluorescence of cardiac tissue from young and old WT and NLRP3−/− mice. Orange arrowheads indicate PCM1+ cardiomyocytes. White arrowheads indicate PCM1− noncardiomyocytes. Dashed lines encircle telomere signals in those cardiac cells. Bars, 10 µm. (b) Telomere length distribution in total cardiac cells (left) and cardiomyocytes (right) of young and old WT and NLRP3−/− mice. Vertical lines indicate mean length (***p < .001, Wilconxon's rank‐sum test)

In order to evaluate the role of NLRP3 during aging of the heart, several markers and pathways associated with aging were studied in hearts from young and aged WT and NLRP3−/− mice. Telomeres in young animals were similar in WT and NLRP3 −/− mice, whereas in two‐year‐old animals, NLRP3 −/− mice showed slightly longer telomeres (Figure 3a,b). This is due to an increased telomere length reduction rate in WT mice compared with NLRP3 −/− mice (Figure S5a). Furthermore, less lipofuscin accumulation in the heart of NLRP3−/− mice was shown after a qualitative observation (Figure S5b). Additionally, we explored classical senescence biomarkers such as IL‐6, p21, and p53. Similar to serum levels of IL‐6, cardiac tissues showed increased IL‐6 protein levels in old mice, accompanied by increased p21 and phospho‐p53 protein levels compared with young mice in WT and KO mice; however, there was a higher increase in old KO mice than WT mice (Figure S6).

**Figure 4:**
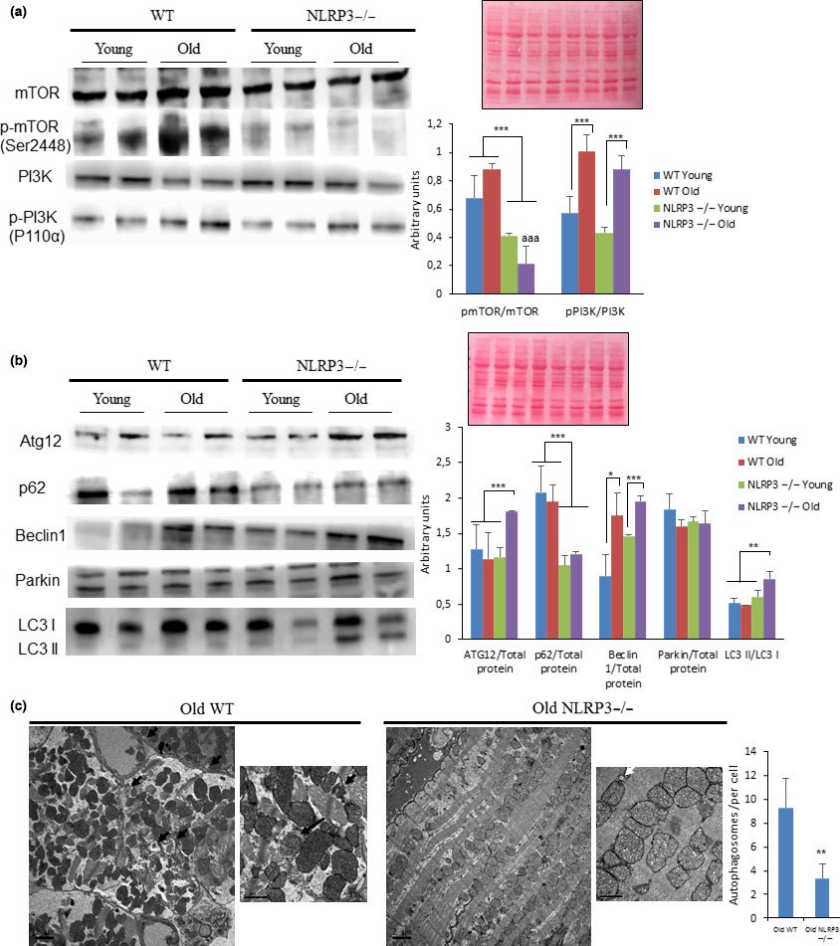
Changes in the Pi3K/mTOR pathways and autophagy observed in cardiac tissues from young and old mice. (a) Western blot analysis showing reduced levels in the Pi3K/mTOR pathway in the heart of NLRP3 −/− mice compared with WT. Densitometric analysis are presented as means ± *SEM*, *n* = 10 mice; ***p < .001 young vs. old mice. ^aaa^ *p* < .001, WT vs. NLRP3 −/− mice. (b) Western blot analysis with representative blot including ATG12, Beclin 1, LC3, Parkin, and p62 level in the heart of Young and old mice. Densitometric analysis are presented as means ± *SEM*, *n* = 10 mice; *p < .05, **p < .005, and ***p < .001 young vs. old mice. (c) Cardiac tissues showing typical ultrastructure with several lamellar bodies and autophagosome (black arrows) present in cardiac tissues from old mice and (white arrows). Scale bar 2 µm (low magnification) and 1 µm (high magnification)

To gain insight into metabolic “longevity regulatory” pathways, we investigated IGF‐1, PI3K, mTOR in the heart. Since NLRP3−/− mice showed low levels of IGF‐1 in young and old mice, we examined signaling changes through these pathways in the heart. Despite no significant differences in phosphorylation of PI3K (p110α), mTOR (Ser2448) was decreased in the heart of aged NLRP3−/− mice (Figure 4a). These data are consistent with the previous observations that cardiac aging is retarded and that healthspan is increased by mTOR inhibition (Inuzuka et al., 2009; Wu et al., 2013). mTOR inhibition is associated with the important physiological process of lysosomal‐dependent recycling, known as autophagy, which is involved in cellular homeostasis through protein degradation and removal of damaged intracellular organelles (Pyo et al., 2013). Autophagic disfunction has also been linked to aging with blocked autophagic flux and accumulation of nondegradated substrates in the form of autophagosome (Pyo et al., 2013). Interestingly, NLRP3−/− mice showed increased levels of ATG12, beclin 1 expression, and LC3II protein expression in NLRP3−/− old mice, with a reduction of p62/SQSTM1 (Figure 4b). From electron microscopic analysis, we corroborated that the numbers of accumulated autophagosomes were reduced in hearts from NLRP3−/− old mice (Figure 4c). This could be explained by where NLRP3 inhibition induced improved autophagy quality in the heart during aging.

### 2.4 Age‐associated change of the cardiac gene expression profile was prevented in NLRP3 −/− mice

**Figure 5:**
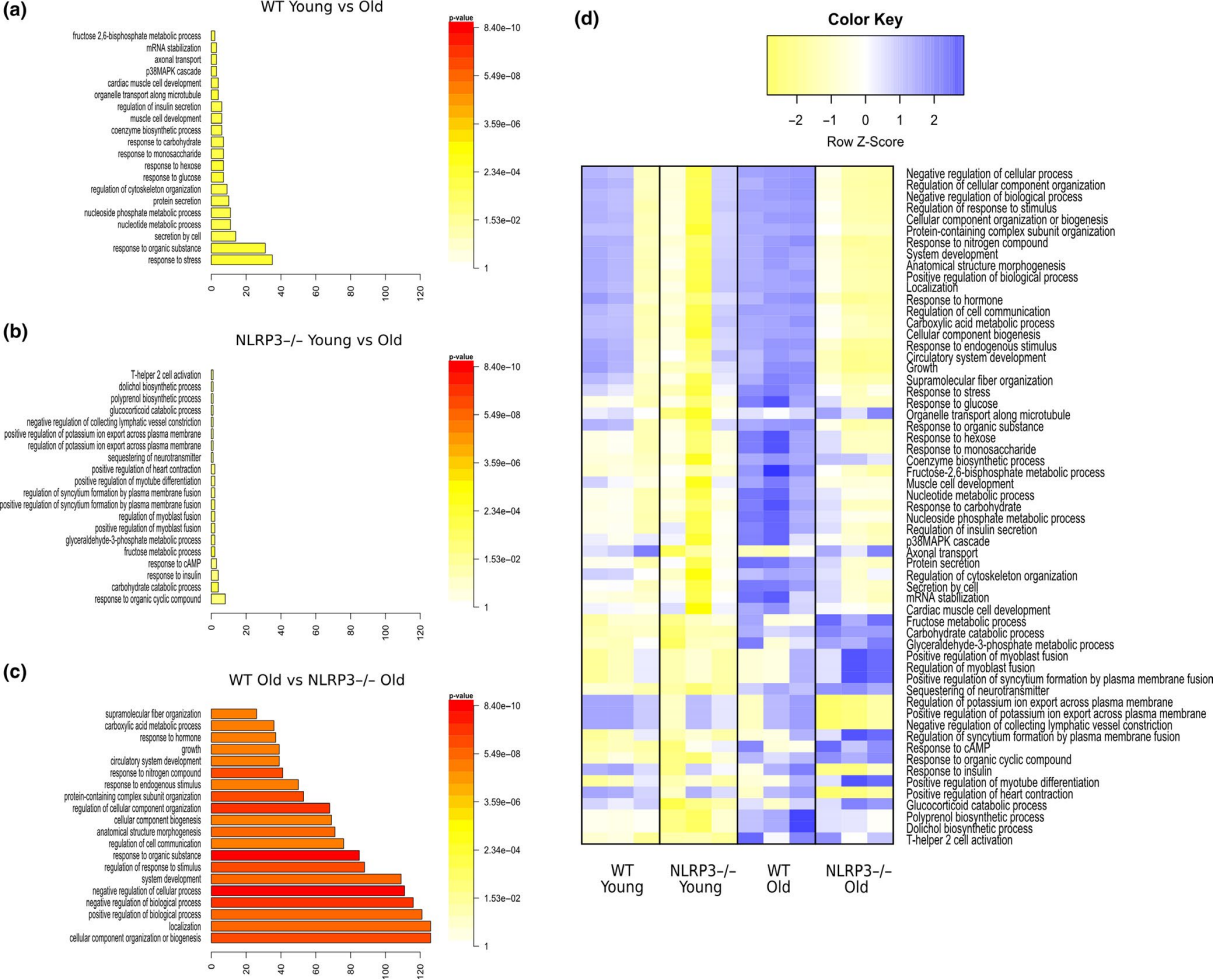
Transcriptional changes in heart from Young and old WT and NLRP3 −/− mice. Gene Ontology enrichment analysis in “Biological Process” in WT young vs. old mice (a), NLRP3 −/− Young vs. old mice (b), and WT old vs. NLRP3 −/− old mice (c). (d) Heatmap clustering of enrichment (z‐scores) of the functions defined by the DAVID in set of coding genes differentially expressed between WT old vs. NLRP3 −/− old mice (*n* = 3 per treatment)

To better define the molecular basis of improved cardiac health in the absence of NLRP3, a microarray expression profiling was performed on cardiac tissues obtained from 22‐month‐old animals. In old WT mice, 202 transcripts (from the 65,956 transcripts examined) changed significantly when compared with old NLRP3−/− mice: 142 transcripts were upregulated and 60 transcripts were downregulated, as those with a fold change equal to or higher than 2, and a p‐value equal to or lower than 0.05 (Figure S7a). The most significant changes common to aging in WT and NLRP3−/− mice are available at ://www.ncbi.nlm.nih.gov/geo/ with code http://www.ncbi.nlm.nih.gov/geo/query/acc.cgi?acc=GSE124483. All the gene expression data were loaded into DAVID for gene ontology (GO) enrichment analysis. The enrichment analysis in WT young‐to‐old mice showed that for the biological process, most of the genes were enriched in the response to stress and organic substances. However, these and other important biological and molecular enriched processes were not significantly different from those in NLRP3−/− young and old mice. NLRP3 deletion conferred protection related to aging, and significant differences were found between WT and NLRP3−/− (Figure 5a–c). To examine the differences in gene expression profiling, gene coding pathways were represented in a heatmap (Figure 5d). Our analysis indicated that 43 pathways were significantly altered between old WT and old NLRP3−/− mice. For a deeper analysis, the downregulated changes in protein coding are presented in Table S3 and the upregulated changes are summarized in Table S4. A subset of expression changes was verified by polymerase chain reaction with reverse transcription (RT‐qPCR) (Figure S8). Notably, the most significant downregulated gene expression in old WT mice compared with old NLRP3 was nicotinamide phosphoribosyltransferase (Nampt), the rate‐limiting enzyme in mammalian NAD^+^ biosynthesis (Table S2). NAD^+^ deficiency is suggested to be a common central pathological factor in a number of diseases, including cardiovascular diseases and aging (North & Sinclair, 2012; Zhang & Ying, 2018). Interestingly, Nampt‐mediated NAD^+^ deficiency is severely associated with glucose intolerance and insulin resistance in metabolic dysfunction by a high‐fat diet (HFD) and aging (Yoshino, Mills, Yoon, & Imai, 2011). Accordingly, we determined NAD^+^ levels from the heart during HFD and a high sucrose diet (HSD) (exposed 15 weeks) and aging in WT and NLRP3−/− mice. In this respect, NLRP3−/− mice showed increased NAD^+^ levels in both cases (HFD, HSD, and aging) and increased SIRT‐1 protein expression (Figure S9A,B). These findings could explain the improved metabolic status and autophagic flux observed in NLRP3−/− mice during aging (Fang et al., 2017).

The homocysteine inducible ER protein with ubiquitin‐like domain 1 (Herpud1), which has been proposed as necessary for adequate insulin‐induced glucose uptake (Navarro‐Marquez et al., 2018), was also shown to be downregulated in old WT when compared to old NLRP3 −/− mice (Table S2). Herpud1 inhibition has recently been associated with induced pathological cardiac hypertrophy, which could explain the reduced hypertrophy observed in NLRP3 −/− mice (Torrealba et al., 2017). Similarly, our transcriptomic study showed upregulated gene expression of the cardiac hypertrophy‐related gene. Expression of established biomarkers atrial natriuretic peptide A (Nppa) and B (Nppb), which are associated with cardiac hypertrophy and strongly upregulated in the ventricular myocardium during cardiac stress (Man, Barnett, & Christoffels, 2018; Newman, Nguyen, Watson, Hull, & Yu, 2017), significantly increased in old WT mice when compared with old NLRP3 mice. Acta1 was the second most upregulated gene in old WT mice when compared with old NLRP3−/− mice. Recently, Acta1 has been associated with cardiac hypertrophy through increased levels of IGF‐1, so the reduced levels of Acta1 in NLRP3−/− mice could be associated with the reduced serum levels of IGF‐1 shown in this study (Bisping et al., 2012). The insulin receptor substrate protein 1 (IRS‐1) was also upregulated in old WT mice when compared to old NLRP3−/− mice. IRS‐1 may contribute to longevity (Selman, Partridge, & Withers, 2011).

Rad (Ras associated with diabetes) GTPase has been established as an endogenous regulator of cardiac excitation‐contraction (Wang et al., 2010). Rrad gene expression was increased in WT, but not in old NLRP3−/− mice, which could explain the increased cardiac pathology. Moreover, upregulation of Thbs1 was also observed in old WT mice when compared with NLRP3−/− mice, which is associated with a disturbed flow from arterial stiffening (Kim et al., 2017). Tumor necrosis factor receptor superfamily member 12a (Tnfrsf12a) and tripartite motif containing 72 (TRIM72) overexpression have been associated with atherosclerosis and diabetic cardiomyopathy, respectively (Liu et al., 2015; Lyu et al., 2018).

Accordingly, these genes were upregulated in old WT mice compared with NLRP3−/− mice, supporting the enhancing role of NLRP3 inhibition in the cardiovascular aging process. Furthermore, the expression of transglutaminase 2 (TGM2), an arterial calcification‐related protein that is positively associated with hypertension and atherosclerosis (Mattison et al., 2014), and Collagen type IV alpha1 (COL4A1) and alpha2 (COL4A2) linked to the pathogenesis of vascular lesions were increased in WT but not in NLRP3−/− mice (Jeanne, Jorgensen, & Gould, 2015). Collectively, these data support significant protection imparted by NLRP3 inhibition on cardiac aging and age‐induced stress and vascular changes that occur during aging. Furthermore, this microarray study showed upregulation of genes associated with the mTOR pathway (Arntl, Akt1 and Ddit4) from old WT mice and changes associated with the negative regulation of autophagy processes (Nampt, Stat3, Fez2 and Akt1), which are associated with our findings of an inhibited mTOR pathway and increased autophagy in NLRP3 −/− mice during aging (Table S2 and S3).

Several of the metabolic changes presented in this study corroborated a special protection for cardiac changes by NLRP3−/− deletion. The metabolic hallmarks related to aging such as glucose tolerance and lipid metabolism are potentially corrected in NLRP3−/− mice, probably related to the reduced IGF‐1 signaling and the PI3K/AKT/mTOR pathway. Notably, adiponectin was also increased in these mice during aging. Adiponectin has been shown to have beneficial cardiovascular effects and to signal through the adiponectin receptors, AdipoR1 and AdipoR2 (Lindgren et al., 2013). AdipoR2−/− mice were resistant to obesity induced by a high‐fat diet and exhibited improved glucose tolerance and decreased plasma cholesterol levels (Fontana, Vinciguerra, & Longo, 2012). Increased levels of AdipoR2 were observed in old WT mice when compared with NLRP3−/−, which could be associated with AdipoR2 deficiency‐dependent protection of atherosclerosis (Lindgren et al., 2013). Furthermore, the reduced levels of adiponectin associated with increased levels of AdipoR2 in WT mice in aging could be interpreted as an attempt to contribute to the optimization of their metabolic environment. This has a negative effect, increasing the leptin/adiponectin ratio.

## 3 DISCUSSION

Aging is the principal pathological process of cardiovascular diseases in healthy people. The principal age‐dependent changes in cardiac structure and function in the heart during normal aging are not well understood and, if defined, could provide new clues for protection from aging‐specific cardiac functional decline. This study showed that NLRP3 is associated with aging by an improved lifespan and healthspan via the modification of several hallmarks of aging. Little has been studied about the role of NLRP3 inhibition during aging, and nothing has been studied about longevity; our data, such as glucose tolerance, are consistent with previous studies on the effect of NLRP3 ablation on aging (Youm et al., 2013). Inflammation is highly associated with aging and age‐related diseases and many rejuvenation strategies adopt anti‐inflammatory diets (Finkel, 2015; Fontana et al., 2012). Increased systemic inflammation is commonly concomitant with metabolic alterations and the deterioration of metabolic health, including the appearance of increased adiposity, insulin resistance, and dyslipidemia, which could prove to be a key determinant of a shortening lifespan and healthspan (Finkel, 2015). According to this, one should anticipate that an experimental manipulation of a specific inflammatory pathway would entail systemic and metabolic effects with an improvement in life expectancy and health. Our results provide convincing evidence that the NLRP3 ablation causes an increase in longevity that could be due to several of the metabolic changes induced by this manipulation. In this study, we have observed an increase in glucose tolerance, a reduction and an increase, respectively, in lectin and adiponectin levels, and a regulation of dyslipemia. All these changes are associated with common pathways, such as IGF‐1, PI3K/AKT/mTOR, autophagy, and intracellular NAD^+^ levels (Finkel, 2015). According to our data, the ablation of NLRP3 showed low serum levels of IGF‐1 in old mice. The role of the protective pathological effects of IGF‐1 is contradictory, but our data suggest that low serum levels of IGF‐1 are the end product of decreased insulin/IGF‐1 signaling, which is known to prolong life, both in invertebrates and in vertebrates (Finkel, 2015; Fontana et al., 2012). Thus, low levels of insulin and/or IGF‐1 signaling, along with a high sensitivity to insulin and IGF‐1, are physiological characteristics that support the prolonged lifespan of Ames dwarf mice (Finkel, 2015) in which the levels of IRS‐1 associated with longevity were reduced (Papaconstantinou & Hsieh, 2015). Interestingly, our transcriptomic analysis showed reduced IRS‐1 expression in old NLRP3 mice when compared to old WT mice.

NLRP3 ablation also showed inhibition of PI3K/AKT/mTOR. mTOR is a serine‐threonine kinase that functions as an intracellular energy sensor whose genetic and pharmacological inhibition has been shown to extend life in a wide range of organisms (Cordero, Williams, & Ryffel, 2018; Wu et al., 2013). Since it is known that mTOR induces autophagy, the ablation of NLRP3 also showed, consistent with previous data showing the effect of inhibition of NLRP3 with other stressors, such as a hypercaloric diet, an increase in autophagy during aging (Pavillard et al., 2017). Cardiac aging is characterized by the presence of hypertrophy, fibrosis, and the accumulation of misfolded proteins and dysfunctional mitochondria. Therefore, autophagy and autophagic fluxes generally were reduced in cardiac tissues during aging, and models of loss of murine autophagy function models show an increase in cardiac dysfunction associated with the accumulation of misfolded proteins and dysfunctional organelles. Accordingly, it has been shown that the stimulation of autophagy improves cardiac function by eliminating accumulated cellular content, thus relieving different aging‐associated pathologies in the heart (Shirakabe, Ikeda, Sciarretta, Zablocki, & Sadoshima, 2016). This mechanism could be key to the improvement of longevity and health induced by the inhibition of NLRP3 and the support of many of the strategies to improve the extension of lifespan and healthspan through the use of rapamycin, caloric restriction with metformin or resveratrol, which have two common mechanisms: an improvement in autophagy and NLRP3 inhibition of inflammasome (Cordero et al., 2018). Furthermore, we found a reduced telomere shortening rate in WT mice when compared to NLRP3 −/− mice. Interestingly, reductions in telomere shortening rate, rather than the initial telomere length, have been suggested as a critical variable that determines a species’ lifespan in a wide variety of species, including mice and humans (Canela, Vera, Klatt, & Blasco, 2007; Vera, Bernardes de Jesus, Foronda, Flores, & Blasco, 2012; Whittemore, Vera, Martínez‐Nevado, Sanpera, & Blasco, 2019). We also found that protein levels of senescence/DNA damage markers, such as p21 or p53, measured by Western blot increase with age in WT hearts, but their expression does not vary with age in NLRP3 −/− hearts, which could be related to a reduced DNA damage response activated by dysfunctional telomeres in these animals. Interestingly, DNA damage in dysfunctional telomeres is a key hallmark of cardiomyocytes with a senescent‐like phenotype (Anderson et al., 2019), and clearance of senescence cells in mice alleviates cardiac deterioration with aging (Anderson et al., 2019; Lewis‐McDougall et al., 2019). Similarly, inhibition or deletion of NLRP3 would improve the detrimental effect of senescence cells in the heart, opening the door to a new line of research. In this sense, the NLRP3 inflammasome activation has been shown to promote the aging of the thymus and lead to immunosenescence (Spadaro et al., 2016). Moreover, the term of inflammaging has been nurtured and associated with a low‐grade proinflammatory phenotype that accompanies aging (Latz & Duewell, 2018). The different implications of NLRP3 in metabolism during aging and the protective role of the inhibition of NLRP3 show a relevant role of this in inflammaging. In this respect, NLRP3 −/− mice showed increased inflammatory levels during aging and, despite this, cardiac aging was prevented by NLRP3 deletion. Senescence influences the cellular environment through the secretion of proinflammatory cytokines, proteases, and chemokines called senescence‐associated secretory phenotype (SASP). Since activation of the NLRP3 system is a probable driver of SASP (Latz & Duewell, 2018), our findings could suggest a role of NLRP3 in the senescence phenotype during inflammaging.

Another age‐related mechanism linked to autophagy impairment is the intracellular reduction of NAD^+^. NAD^+^ is an electron acceptor in the mitochondrial electron transport chain that is also an essential substrate for NAD^+^‐dependent enzymes, such as sirtuins and poly ADP ribose polymerase (Rajman, Chwalek, & Sinclair, 2018). NAD^+^ levels decrease with age due to Nampt downregulation, oxidative stress, inflammation, defective circadian rhythm, and accumulation of DNA damage. Nampt, a key enzyme in the salvage pathway of NAD^+^ biosynthesis, is downregulated in the heart in response to ischemia, which induces a decrease in NAD^+^ levels in the heart, inhibition of autophagic flux, and cell death (Shirakabe et al., 2016). Therefore, restoring NAD^+^ content by overexpressing Nampt or adding NAD^+^ supplements restores the level of autophagy during ischemia and reduces the extent of myocardial infarction (Rajman et al., 2018). We observed an increased level of Nampt in NLRP3−/− old mice by transcriptomic analysis, which was corroborated by real‐time PCR. This is probably connected to the high levels of NAD^+^ observed in hearts of elderly NLRP3−/− mice fed with hypercaloric diets.

We acknowledge the limitations of using male mice only. On the other hand, we have studied the effect of the NLRP3 ablation in the expression of several markers of senescence by Western blots; however, our results must be extended to detect the specific cell type using, among others, gH2AX‐telomere immuno FISH or gH2AX‐PML colocalization in Immunofluorescence and IHC. Thus, future investigations should account for the implication of other inflammasomes in aging and their modulation.

In conclusion, our findings suggest that NLRP3 inhibition attenuates the harmful effects of cardiac aging and extends the lifespan in male mice. NLRP3 ablation improves metabolic characteristics related to aging, such as glucose tolerance, lipid metabolism, and leptin/adiponectin. These results could be associated with reduced IGF‐1 signaling and the PI3K/AKT/mTOR pathway and with autophagy activation. Our data associate the inhibition of NLRP3 with previous interventions against aging, such as caloric restriction, metformin, resveratrol or protein restriction, and involve levels of Nampt‐dependent NAD^+^ and SIRT1 (Cordero et al., 2018). In addition, our transcriptomic results show a profile related to metabolic improvement and an anti‐hypertrophic effect of cardiac protection. Finally, NLRP3 inhibition could be associated with a specific inflammasome‐dependent inflammaging. Therefore, prevention of the aging process through multiple mechanisms by NLRP3 inhibition is likely to attenuate the associated decrease in cardiac function. Thus, it offers a promising goal for the prevention of cardiac aging.

## 4 EXPERIMENTAL PROCEDURES

### 4.1 Ethical statements

Animal studies were performed in accordance with European Union guidelines (2010/63/EU) and the corresponding Spanish regulations for the use of laboratory animals in chronic experiments (RD 53/2013 on the care of experimental animals). All experiments were approved by the local institutional animal care committee.

### 4.2 Animals

For all experiments, only male mice were used. Young and old NLRP3−/− transgenic mice (C57BL/6J background) and WT/NLRP3+/+ littermate controls, weighing 25–30 g, were maintained on a regular 12 hr light/dark cycle. All groups had ad libitum access to their prescribed diet and water throughout the whole study. Body weight and food intake were monitored weekly. Animal rooms were maintained at 20–22°C with 30%–70% relative humidity.

### 4.3 Mouse longevity study

Young and old C57/BL6/J and NLRP3−/− transgenic mice (C57BL/6J background), weighing 25–30 g, were maintained on a regular 12 hr light/dark cycle. Mice were housed in groups of four to eight same‐sex littermates under specific pathogen‐free conditions. Individuals were monitored daily and weighed monthly, but were otherwise left undisturbed until they died. Survival was assessed using male and female mice, and all animals were dead by the time of this report. Kaplan–Meier survival curves were constructed using known birth and death dates, and differences between groups were evaluated using the logrank test.

### 4.4 Reagents

Monoclonal antibodies specific for Beclin‐1 and p62 were purchased from Sigma‐Aldrich. The anti‐GAPDH monoclonal antibody was acquired from Calbiochem‐Merck Chemicals Ltd. Similarly, anti‐active caspase‐3, anti‐SIRT‐1, p‐PI3K and PI3K, p‐mTOR and mTOR, and anti‐Parkin were obtained from Cell Signaling Technology. Finally, anti‐Bcl‐2, anti‐Bax, anti‐ATG12, and anti‐MAP‐LC3 antibodies were obtained from Santa Cruz Biotechnology. A cocktail of protease inhibitors (Complete™ Protease Inhibitor Cocktail) was purchased from Boehringer Mannheim. The Immun Star HRP substrate kit was obtained from Bio‐Rad Laboratories Inc.

### 4.5 Glucose tolerance test

Glucose tolerance tests were performed by fasting the mice overnight for 16 hr and then injecting glucose (1 g/kg), intraperitoneally. Glucose measurements were performed using a Bayer Contour blood glucose meter and test strips.

### 4.6 Leptin, adiponectin, and IGF‐1

Serum levels of leptin, adiponectin, and IGF‐1 were assayed in duplicate using commercial ELISA kits (R&D Systems).

### 4.7 Serum biomarkers

Serum levels of glucose, triglycerides, cholesterol, uric acid, aspartate aminotransferase, alanine aminotransferase, and creatine kinase were assayed using commercial kits (Randox Laboratories).

### 4.8 Immunoblotting

Western blotting was performed using standard methods. After protein transfer, the membrane was incubated with various primary antibodies diluted at 1:1,000 and then with the corresponding secondary antibodies coupled to horseradish peroxidase at a 1:10,000 dilution. Specific protein complexes were identified using the Immun Star HRP substrate kit (Biorad Laboratories Inc.).

### 4.9 Electrocardiography

Mice were anesthetized with 1.5%–2% isoflurane in oxygen, inhaled through a facial mask. To avoid night–day circadian variations, ECG was performed in the morning. ECG electrodes were inserted subcutaneously in the four limbs, and sequential ECG recordings were acquired at 2 KHz sweep‐speed using a MP36R data acquisition workstation (Biopac Systems). Data were stored for off‐line analysis using custom MatLab scripts. In 3 and 20 months of age/ death of the animal, NLRP3−/− and WT mice were given a weekly β‐adrenergic challenge with isoproterenol (i.v. bolus 0.34 mg/kg). ECG traces were recorded at baseline, after challenge, and during recovery (10–25 min).

ECG recordings were analyzed offline using custom scripts for preprocessing, visualization and quantification of electrophysiologic intervals and heart rate variability markers. After band‐pass filtering between 0.5 and 250 Hz, baseline wander was removed using a bidirectional filtering strategy. Baseline drift removal is essential for morphological analysis of T waves. Specifically: (a) PR intervals were measured from the beginning or the P wave to the peak of the R wave; (b) QRS intervals were measured from the beginning of the Q wave until the point where the S wave crosses the baseline; and (c) QT intervals were measured from the beginning of the Q wave until the point where the T wave declined to 90% (T90) from the peak. Finite differential methods and wavelet transform were used for fiducial point estimation. R‐peak detection was robustly estimated by parabolic fitting of the coiflet wavelet transform and detection of the maximum magnitude point. All R detections were supervised to validate accuracy of ECG segmentations. After QRS detection, P and T wave segments were extracted using adaptive windowing, depending upon beat‐to‐beat RR changes. After segmentation using differential methods, both waves were low‐pass filtered at 20 Hz using a Kaiser window FIR filter.

### 4.10 Echocardiography

Two‐dimensional and M‐mode echocardiography longitudinal studies were performed in 3‐month intervals throughout the mice's entire lifespan. Males (*n* = 10–15) were anesthetized (1.5%–2% isoflurane in a mixture with oxygen), and the analysis was carried out using a Vevo770 system (Vevo 2100, Visualsonics Inc.) equipped with a 30‐MHz linear transducer probe. To avoid night–day circadian variations, echocardiographs were performed in the mornings. Before echocardiography, animal fur was removed with a depilatory agent and animals were warmed to maintain body temperature. The heart was imaged in the 2D parasternal long‐ and short‐axis projections with guided M‐mode recordings at the midventricular level in both views. Images were recorded and transferred to a computer for posterior blinded analysis using the Vevo 2100 Workstation software. LV end‐diastolic diameter (LVEDD), LV‐systolic diameter (LVESD), end‐diastolic LV anterior wall thickness (LVAW), and LV posterior wall thickness (LVPW) were measured from images obtained with M‐mode echocardiography. LV fractional shortening (FS) and aortic cardiac output (AA.CO) was calculated. LV ejection fraction (EF) by the Teichholz formula was assessed by using the M‐mode images displayed over time and obtained from a single line in the middle of LV. The LV mass was calculated from the same M‐mode images by using diastolic LV diameters of LV internal diameter (LVID), posterior wall (PW), and interventricular septum (IVS) as follow: LV Mass (mg) = 1.05 (Lid + LVPWd + IVSd3 – LVIDd3) 0.8. Corrected LV Mass = (LV Mass) 0.8.

### 4.11 Histological study

After anesthesia of mice, hearts were excised and placed in a 16 mM KCl solution to arrest the heart in diastole prior to fixation and immediately placed in 10% neutral‐buffered formalin at room temperature for 24 hr after a brief rinse with PBS. The specimens were embedded in paraffin, cut into 5‐μm sections, and stained with hematoxylin and eosin. To detect fibrosis in heart sections, consecutive formalin‐fixed, paraffin‐embedded, 4‐µm sections were stained with Masson trichrome or with Sirius Red. Masson trichrome staining was performed following the manufacturer's instructions (Accustain HT15, Sigma‐Aldrich). Sirius Red staining was performed by incubating slides in 0.1% Sirius Red F3B for 1 hr, rinsing twice in acidified water, dehydrating thrice in 100% ethanol, and then clearing in xylene. Manually delimited cardiomyocyte or fibrotic areas were also calculated on a digital microscope (×400) with ImageJ (version ImageJ 1.49) software.

### 4.12 Electron microscopy

Transmission electron microscopy (TEM). Mice were euthanized by cervical dislocation and the left ventricle apex was immediately dissected (3 months old, *n* = 3 per genotype and 20 months old, *n* = 3 per genotype). Heart samples were fixed in 2.5% glutaraldehyde and 4% formaldehyde in 0.1 M HEPES buffer for 4–5 hr. After buffer washes, samples were postfixed for 1 hr at room temperature in a 1:1 solution of 1% osmium tetroxide and 3% aqueous potassium ferrocyanide. Samples were rinsed in distilled H_2_O. Tissues were dehydrated through a graded acetone series and embedded in Spurr's low viscosity embedding mixture (Electron Microscopy Sciences). Ultra‐thin sections (60 nm) were then mounted on copper grids and stained with lead citrate. Samples were examined on a JEOL 10‐10 electron microscope through 1,500×, 5,000×, 40,000×, and 80,000× objectives. Mitochondrial morphometry, cristae area (2 images, 5,000×, per animal), and lipid droplets (50 images, 5,000×, per animal) were segmented manually and analyzed using Fiji (http://fiji.sc/Fiji) and ImageJ 1.48v software. The investigator was blinded to the group allocation when assessing the outcome.

### 4.13 Telomere length analysis

The telomere length of cardiac cells was measured by quantitative fluorescence in situ hybridization (Q‐FISH). Hearts were fixed in 4% PFA, and paraffin‐embedded tissue sections of the same thickness (4 μm) were first immunostained for PCM1 (1.300). Slides were then fixed in 4% formaldehyde for 2 min, treated with 0.28 mM pepsin for 10 min at 37°C and then hybridized with a Cy3‐labeled peptide nucleic acid probe targeting the telomere repeat sequence (Flores et al., 2008). The intensity of the fluorescent signal for a given telomere is directly proportional to telomere length, providing a quantitative measure of telomere length (Lansdorp et al., 1996).

For telomere length analysis, DAPI, Cy3 (telomeres), and immunofluorescence signals were acquired sequentially in separate channels of a confocal microscope (SP5) fitted with a 63× objective and linked to LasAF and MatrixScreener software. All slides were stained at the same time, and all images were acquired at the same laser intensity. For image quantification, Nikon NIS Elements software was used to subtract Cy3 channel background and to generate maximum‐intensity projections from 16‐bit image stacks (15 sections at steps of 1.0 µm). Quantitative image analysis was performed using the Metamorph platform as previously described (Flores et al., 2008). The DAPI images were signal‐intensity thresholded. After conversion to a 1‐bit binary mask, the DAPI image was used to define the nuclear area and the Cy3 image to quantify telomere fluorescence. The binary DAPI mask was applied to the Cy3 image to obtain a combined image with the telomere fluorescence information for each nucleus. Cy3 fluorescence intensity was measured as the mean gray value in each nucleus in arbitrary units of fluorescence (auf). Telomere intensity values were exported to Excel. For the determination of telomere length in cardiomyocytes, cardiomyocyte nuclei were manually selected with Metamorph using the PCM1 immunofluorescence images. Only cells clearly identifiable as cardiomyocytes were considered for the analysis.

### 4.14 Transcriptome analysis

We used the Affymetrix clariom^TM^ D assay mouse, which includes 65,956 genes. The raw data were analyzed by the Affymetrix software Trancriptome Analysis Console. Expression values were normalized by SST (Signal Space Transformation)‐RMA (Robust Microarray Analysis; Irizarry et al., 2003), and the mutant mice were compared vs. WT, using the 3 replicates for every analysis. Then, a fold change and p‐value were calculated for every gene by an unpaired test one‐way (single factor) using the NMATH package. A gene was considered as differentially expressed when it had a fold change equal to or higher than 1.5, and a p‐value equal to or lower than 0.05 using Fisher's exact test. Then, a functional enrichment was performed with the differentially expressed genes using the functional annotation tool from the DAVID web site (https://www.ncbi.nlm.nih.gov/pmc/articles/PMC3381967/). As such, we found a number of annotation categories. To analyze the expression profile for the genes included in these categories, a heatmap was created for every annotation category, using the heatmap.2 function in the gplots R package, and considering normalized values by z‐scores, and making a clustering based on the expression profiles and the average method. Finally, a mean of the expression values in every annotation category was calculated and it was again plotted in a heatmap.

### 4.15 Quantification of intracellular NAD^+^

Total NAD^+^ concentrations were determined independently in whole cardiac extracts using the NAD^+^ cycling assay (Abcam ab65348, Inc.), as described previously. NAD^+^ levels were expressed as pmol/mgprotein.

### 4.16 Statistics

All data are expressed as means ± *SEM*. After, evaluation of normality using the Shapiro–Wilk test, statistical differences among the different groups were measured using either an unpaired Student *t* test or 1‐way analysis of variance (ANOVA) when appropriate with Tukeys post hoc test. A Wilcoxon's ram sum test was used to calculate the statistical significance between telomere length distributions. A *p*‐value of ≤.05 was considered statistically significant. Statistical analyses were performed using Prism software version 5.0a (GraphPad). Asterisks in the figures represent the following: *: *p* ≤ .05; **: *p* ≤ .01; and ***: *p* ≤ .001.

## CONFLICT OF INTERESTS

MAC currently holds a fractional Professorial Research Fellow appointment at the University of Queensland with his remaining time as CEO of Inflazome Ltd., a company headquartered in Dublin, Ireland that is developing drugs to address unmet clinical needs in inflammatory disease by targeting the inflammasome. AR is an inventor on inflammasome related patents (WO2017140778 and WO2016131098).

## Data Availability

Results of transcriptomic changes by microarray and most significant changes common to aging in WT and NLRP3−/− mice are available at http://www.ncbi.nlm.nih.gov/geo/ with code http://www.ncbi.nlm.nih.gov/geo/query/acc.cgi?acc=GSE124483.

## References

[acel13050-bib-0001] Anderson, R. , Lagnado, A. , Maggiorani, D. , Walaszczyk, A. , Dookun, E. , Chapman, J. , … Passos, J. F. . (2019). Length‐independent telomere damage drives post‐mitotic cardiomyocyte senescence. The EMBO Journal, 38 pii: e100492. 10.15252/embj.2018100492 PMC6396144 30737259

[acel13050-bib-0002] Bisping, E. , Ikeda, S. , Sedej, M. , Wakula, P. , McMullen, J. R. , Tarnavski, O. , … Pieske, B. (2012). Transcription factor GATA4 is activated but not required for insulin‐like growth factor 1 (IGF1)‐induced cardiac hypertrophy. Biological Chemistry, 287, 9827–9834. 10.1074/jbc.M111.338749 PMC3323002 22228770

[acel13050-bib-0003] Brandhorst, S. , Choi, I. Y. , Wei, M. , Cheng, C. W. , Sedrakyan, S. , Navarrete, G. , … Longo, V. D. (2015). A periodic diet that mimics fasting promotes multi‐system regeneration, enhanced cognitive performance, and healthspan. Cell Metabolism, 22, 86–99. 26094889 10.1016/j.cmet.2015.05.012 PMC4509734

[acel13050-bib-0004] Bullón, P. , Cano‐García, F. J. , Alcocer‐Gómez, E. , Varela‐López, A. , Roman‐Malo, L. , Ruiz‐Salmerón, R. J. , … Cordero, M. D. (2017). Could NLRP3‐inflammasome be a cardiovascular risk biomarker in acute myocardial infarction patients? Antioxidants & Redox Signaling, 27, 269–275. 27967213 10.1089/ars.2016.6970

[acel13050-bib-0005] Canela, A. , Vera, E. , Klatt, P. , & Blasco, M. A. (2007). High‐throughput telomere length quantification by FISH and its application to human population studies. Proceedings of the National Academy of Sciences of the United States of America, 104, 5300–5305. 17369361 10.1073/pnas.0609367104 PMC1828130

[acel13050-bib-0006] Cordero, M. D. , Williams, M. R. , & Ryffel, B. (2018). AMP‐activated protein kinase regulation of the NLRP3 inflammasome during aging. Trends in Endocrinology and Metabolism, 29, 8–17. 29150317 10.1016/j.tem.2017.10.009

[acel13050-bib-0007] DiNicolantonio, J. J. , Lucan, S. C. , & O'Keefe, J. H. (2016). The evidence for saturated fat and for sugar related to coronary heart disease. Progress in Cardiovascular Diseases, 58, 464–472. 26586275 10.1016/j.pcad.2015.11.006 PMC4856550

[acel13050-bib-0008] Fang, E. F. , Lautrup, S. , Hou, Y. , Demarest, T. G. , Croteau, D. L. , Mattson, M. P. , & Bohr, V. A. (2017). NAD+ in aging: Molecular mechanisms and translational implications. Trends in Molecular Medicine, 23, 899–916. 28899755 10.1016/j.molmed.2017.08.001 PMC7494058

[acel13050-bib-0009] Finkel, T. (2015). The metabolic regulation of aging. Nature Medicine, 21, 1416–1423. 10.1038/nm.3998 26646498

[acel13050-bib-0010] Flores, I. , Canela, A. , Vera, E. , Tejera, A. , Cotsarelis, G. , & Blasco, M. A. (2008). The longest telomeres: A general signature of adult stem cell compartments. Genes & Development, 22, 654–667. 18283121 10.1101/gad.451008 PMC2259034

[acel13050-bib-0011] Fontana, L. , Vinciguerra, M. , & Longo, V. D. (2012). Growth factors, nutrient signaling, and cardiovascular aging. Circulation Research, 110, 1139–1150. 22499903 10.1161/CIRCRESAHA.111.246470 PMC3376758

[acel13050-bib-0012] Ford, E. S. , Li, C. , Zhao, G. , Pearson, W. S. , & Capewell, S. (2009). Trends in the prevalence of low risk factor burden for cardiovascular disease among United States adults. Circulation, 120, 1181–1208. 19752328 10.1161/CIRCULATIONAHA.108.835728

[acel13050-bib-0013] Gómez‐Pardo, E. , Fernández‐Alvira, J. M. , Vilanova, M. , Haro, D. , Martínez, R. , Carvajal, I. , … Fuster, V. (2016). A comprehensive lifestyle peer group‐based intervention on cardiovascular risk factors: The randomized controlled fifty‐fifty program. Journal of the American College of Cardiology, 67, 476–485. 26562047 10.1016/j.jacc.2015.10.033

[acel13050-bib-0014] Inuzuka, Y. , Okuda, J. , Kawashima, T. , Kato, T. , Niizuma, S. , Tamaki, Y. , … Shioi, T. (2009). Suppression of phosphoinositide 3‐kinase prevents cardiac aging in mice. Circulation, 120, 1695–1703. 19822807 10.1161/CIRCULATIONAHA.109.871137

[acel13050-bib-0015] Irizarry, R. A. , Bolstad, B. M. , Collin, F. , Cope, L. M. , Hobbs, B. , & Speed, T. P. (2003). Summaries of Affymetrix GeneChip probe level data. Nucleic Acids Research, 31(4), e15. 12582260 10.1093/nar/gng015 PMC150247

[acel13050-bib-0016] Jeanne, M. , Jorgensen, J. , & Gould, D. B. (2015). Molecular and genetic analyses of collagen type IV mutant mouse models of spontaneous intracerebral hemorrhage identify mechanisms for stroke prevention. Circulation, 131, 1555–1565. 25753534 10.1161/CIRCULATIONAHA.114.013395 PMC4497509

[acel13050-bib-0017] Kim, C. W. , Pokutta‐Paskaleva, A. , Kumar, S. , Timmins, L. H. , Morris, A. D. , Kang, D. W. , … Brewster, L. P. (2017). Disturbed flow promotes arterial stiffening through thrombospondin‐1. Circulation, 136, 1217–1232. 28778947 10.1161/CIRCULATIONAHA.116.026361 PMC5614852

[acel13050-bib-0018] Lansdorp, P. M. , Verwoerd, N. P. , van de Rijke, F. M. , Dragowska, V. , Little, M. T. , Dirks, R. W. , … Tanke, H. J. (1996). Heterogeneity in telomere length of human chromosomes. Human Molecular Genetics, 5, 685–691. 8733138 10.1093/hmg/5.5.685

[acel13050-bib-0019] Latz, E. , & Duewell, P. (2018). NLRP3 inflammasome activation in inflammaging. Seminars in Immunology, 40, 61–73. 30268598 10.1016/j.smim.2018.09.001

[acel13050-bib-0020] Lewis‐McDougall, F. C. , Ruchaya, P. J. , Domenjo‐Vila, E. , Shin Teoh, T. , Prata, L. , Cottle, B. J. , … Ellison‐Hughes, G. M. (2019). Aged‐senescent cells contribute to impaired heart regeneration. Aging Cell, 18, e12931. 30854802 10.1111/acel.12931 PMC6516154

[acel13050-bib-0021] Lindgren, A. , Levin, M. , Rodrigo Blomqvist, S. , Wikström, J. , Ahnmark, A. , Mogensen, C. , … Linden, D. (2013). Adiponectin receptor 2 deficiency results in reduced atherosclerosis in the brachiocephalic artery in apolipoprotein E deficient mice. PLoS ONE, 8, e80330. 24324556 10.1371/journal.pone.0080330 PMC3855811

[acel13050-bib-0022] Liu, D. , Zeng, X. , Li, X. , Mehta, J. L. , & Wang, X. (2017). Role of NLRP3 inflammasome in the pathogenesis of cardiovascular diseases. Basic Research in Cardiology, 113, 5. 29224086 10.1007/s00395-017-0663-9

[acel13050-bib-0023] Liu, F. , Song, R. , Feng, Y. , Guo, J. , Chen, Y. , Zhang, Y. , … Xiao, R. P. (2015). Upregulation of MG53 induces diabetic cardiomyopathy through transcriptional activation of peroxisome proliferation‐activated receptor α. Circulation, 131, 795–804. 25637627 10.1161/CIRCULATIONAHA.114.012285

[acel13050-bib-0024] Lyu, M. , Cui, Y. , Zhao, T. , Ning, Z. , Ren, J. , Jin, X. , … Zhu, Y. (2018). Tnfrsf12a‐mediated atherosclerosis signaling and inflammatory response as a common protection mechanism of shuxuening injection against both myocardial and cerebral ischemia‐reperfusion injuries. Frontiers in Pharmacology, 9, 312. 29681850 10.3389/fphar.2018.00312 PMC5897438

[acel13050-bib-0025] Magnani, J. W. , Wang, N. , Nelson, K. P. , Connelly, S. , Deo, R. , Rodondi, N. , … Benjamin, E. J. (2013). Health, Aging, and body composition study. Electrocardiographic PR interval and adverse outcomes in older adults: The health, aging, and body composition study. Circulation. Arrhythmia and Electrophysiology, 6, 84–90. 23243193 10.1161/CIRCEP.112.975342 PMC3613778

[acel13050-bib-0026] Man, J. , Barnett, P. , & Christoffels, V. M. (2018). Structure and function of the Nppa‐Nppb cluster locus during heart development and disease. Cellular and Molecular Life Sciences, 75, 1435–1444. 29302701 10.1007/s00018-017-2737-0 PMC5852170

[acel13050-bib-0027] Mattison, J. A. , Wang, M. , Bernier, M. , Zhang, J. , Park, S. S. , Maudsley, S. , … de Cabo, R. (2014). Resveratrol prevents high fat/sucrose diet‐induced central arterial wall inflammation and stiffening in nonhuman primates. Cell Metabolism, 20, 183–190. 24882067 10.1016/j.cmet.2014.04.018 PMC4254394

[acel13050-bib-0028] McBride, M. J. , Foley, K. P. , D'Souza, D. M. , Li, Y. E. , Lau, T. C. , Hawke, T. J. , & Schertzer, J. D. (2017). The NLRP3 inflammasome contributes to sarcopenia and lower muscle glycolytic potential in old mice. American Journal of Physiology. Endocrinology and Metabolism, 313, E222–E232. 28536183 10.1152/ajpendo.00060.2017 PMC5582883

[acel13050-bib-0029] Navarro‐Marquez, M. , Torrealba, N. , Troncoso, R. , Vásquez‐Trincado, C. , Rodriguez, M. , Morales, P. E. , … Lavandero, S. (2018). Herpud1 impacts insulin‐dependent glucose uptake in skeletal muscle cells by controlling the Ca2+‐calcineurin‐Akt axis. Biochimica Et Biophysica Acta, 1864, 1653–1662. 29486284 10.1016/j.bbadis.2018.02.018

[acel13050-bib-0030] Newman, M. S. , Nguyen, T. , Watson, M. J. , Hull, R. W. , & Yu, H. G. (2017). Transcriptome profiling reveals novel BMI‐ and sex‐specific gene expression signatures for human cardiac hypertrophy. Physiological Genomics, 49, 355–367. 28500252 10.1152/physiolgenomics.00122.2016 PMC5538878

[acel13050-bib-0031] North, B. J. , & Sinclair, D. A. (2012). The intersection between aging and cardiovascular disease. Circulation Research, 110, 1097–1108. 22499900 10.1161/CIRCRESAHA.111.246876 PMC3366686

[acel13050-bib-0032] Papaconstantinou, J. , & Hsieh, C. C. (2015). IGF‐1 mediated phosphorylation of specific IRS‐1 serines in Ames dwarf fibroblasts is associated with longevity. Oncotarget., 6, 35315–35323. 26474286 10.18632/oncotarget.6112 PMC4742107

[acel13050-bib-0033] Pavillard, L. E. , Cañadas‐Lozano, D. , Alcocer‐Gómez, E. , Marín‐Aguilar, F. , Pereira, S. , Robertson, A. A. B. , … Cordero, M. D. (2017). NLRP3‐inflammasome inhibition prevents high fat and high sugar diets‐induced heart damage through autophagy induction. Oncotarget, 8, 99740–99756. 29245937 10.18632/oncotarget.20763 PMC5725128

[acel13050-bib-0034] Pyo, J. O. , Yoo, S. M. , Ahn, H. H. , Nah, J. , Hong, S. H. , Kam, T. I. , … Jung, Y. K. (2013). Overexpression of Atg5 in mice activates autophagy and extends lifespan. Nature Communications, 4, 2300. 10.1038/ncomms3300 PMC3753544 23939249

[acel13050-bib-0035] Rajman, L. , Chwalek, K. , & Sinclair, D. A. (2018). Therapeutic potential of NAD‐boosting molecules: The in vivo evidence. Cell Metabolism, 27, 529–547. 29514064 10.1016/j.cmet.2018.02.011 PMC6342515

[acel13050-bib-0036] Selman, C. , Partridge, L. , & Withers, D. J. (2011). Replication of extended lifespan phenotype in mice with deletion of insulin receptor substrate 1. PLoS ONE, 6, e16144. 21283571 10.1371/journal.pone.0016144 PMC3026792

[acel13050-bib-0037] Shirakabe, A. , Ikeda, Y. , Sciarretta, S. , Zablocki, D. K. , & Sadoshima, J. (2016). Aging and autophagy in the heart. Circulation Research, 118, 1563–1576. 27174950 10.1161/CIRCRESAHA.116.307474 PMC4869999

[acel13050-bib-0038] Spadaro, O. , Goldberg, E. L. , Camell, C. D. , Youm, Y. H. , Kopchick, J. J. , Nguyen, K. Y. , … Dixit, V. D. (2016). Growth hormone receptor deficiency protects against age‐related NLRP3 inflammasome activation and immune senescence. Cell Reports, 14, 1571–1580. 26876170 10.1016/j.celrep.2016.01.044 PMC5992590

[acel13050-bib-0039] Torrealba, N. , Navarro‐Marquez, M. , Garrido, V. , Pedrozo, Z. , Romero, D. , Eura, Y. , … Lavandero, S. (2017). Herpud1 negatively regulates pathological cardiac hypertrophy by inducing IP3 receptor degradation. Scientific Reports, 7, 13402. 29042597 10.1038/s41598-017-13797-z PMC5645377

[acel13050-bib-0040] Vera, E. , Bernardes de Jesus, B. , Foronda, M. , Flores, J. M. , & Blasco, M. A. (2012). The rate of increase of short telomeres predicts longevity in mammals. Cell Reports, 2, 732–737. 23022483 10.1016/j.celrep.2012.08.023

[acel13050-bib-0041] Wang, G. , Zhu, X. , Xie, W. , Han, P. , Li, K. , Sun, Z. , … Cheng, H. (2010). Rad as a novel regulator of excitation‐contraction coupling and beta‐adrenergic signaling in heart. Circulation Research, 106, 317–327. 19926875 10.1161/CIRCRESAHA.109.208272

[acel13050-bib-0042] Whittemore, K. , Vera, E. , Martínez‐Nevado, E. , Sanpera, C. , & Blasco, M. A. (2019). Telomere shortening rate predicts species life span. Proceedings of the National Academy of Sciences of the United States of America, 116, 15122–15127. 31285335 10.1073/pnas.1902452116 PMC6660761

[acel13050-bib-0043] Wu, J. J. , Liu, J. , Chen, E. B. , Wang, J. J. , Cao, L. , Narayan, N. , … Finkel, T. (2013). Increased mammalian lifespan and a segmental and tissue‐specific slowing of aging after genetic reduction of mTOR expression. Cell Reports, 4, 913–920. 23994476 10.1016/j.celrep.2013.07.030 PMC3784301

[acel13050-bib-0044] Yoshino, J. , Mills, K. F. , Yoon, M. J. , & Imai, S. (2011). Nicotinamide mononucleotide, a key NAD(+) intermediate, treats the pathophysiology of diet‐ and age‐induced diabetes in mice. Cell Metabolism, 14, 528–536. 21982712 10.1016/j.cmet.2011.08.014 PMC3204926

[acel13050-bib-0045] Youm, Y. H. , Grant, R. W. , McCabe, L. R. , Albarado, D. C. , Nguyen, K. Y. , Ravussin, A. , … Dixit, V. D. (2013). Canonical Nlrp3 inflammasome links systemic low‐grade inflammation to functional decline in aging. Cell Metabolism, 18, 519–532. 24093676 10.1016/j.cmet.2013.09.010 PMC4017327

[acel13050-bib-0046] Zhang, M. , & Ying, W. (2018). NAD+ deficiency is a common central pathological factor of a number of diseases and aging: Mechanisms and therapeutic implications. Antioxidants & Redox Signaling, 30, 890–905. 29295624 10.1089/ars.2017.7445

