# Supplementary material for: NLRP3 inflammasome suppression improves longevity and prevents cardiac aging in male mice
Source: Aging Cell. 2019 Oct 18;19(1):e13050. doi: 10.1111/acel.13050 (PMC6974709; doi:10.1111/acel.13050)
Supplement: Supplementary file 1 [file ACEL-19-e13050-s001.docx]

**SUPPLEMENTARY DATA**

**NLRP3-inflammasome suppression improves longevity and prevents cardiac aging in male mice**

Fabiola Marín-Aguilar^1^, Ana V. Lechuga-Vieco^2,3^, Diego Cañadas-Lozano^1^, Elísabet Alcocer-Gómez^4^, Beatriz Castejón-Vega^1^, Javier Lucas^2^, Carlos Garrido^2^, Alejandro Peralta-Garcia^5^, Antonio J. Pérez-Pulido^5^, Alfonso Varela-López^6^, José L. Quiles^6^, Bernhard Ryffel^7^, Ignacio Flores^2^, Pedro Bullón^1^, Jesús Ruiz-Cabello^3,8,9,10^, Mario D. Cordero^6^

^1^ Research Laboratory, Oral Medicine Department, University of Sevilla, Sevilla, Spain

^2^ Centro Nacional de Investigaciones Cardiovasculares Carlos III (CNIC), 28049 Madrid, Spain; CIBERES: C/ Melchor Fernández-Almagro 3, 28029 Madrid, Spain.

^3^ CIBER de Enfermedades Respiratorias (CIBERES), Madrid, Spain

^4^ Departamento de Psicología Experimental, Facultad de Psicología, Universidad de Sevilla, Seville Spain

^5^ Centro Andaluz de Biología del Desarrollo (CABD), Universidad Pablo de Olavide-CSIC-Junta de Andalucía, Sevilla, Spain.

^6^ Institute of Nutrition and Food Technology "José Mataix Verdú", Department of Physiology, Biomedical Research Center, University of Granada, 18100 Granada, Spain

^7^ Laboratory of Experimental and Molecular Immunology and Neurogenetics (INEM), UMR 7355 CNRS-University of Orleans, Orléans, France; IDM, University of Cape Town, South Africa

^8^ CIC biomaGUNE, San Sebastian-Donostia, Spain

^9^ IKERBASQUE, Basque Foundation for Science, Spain.

^10^ Universidad Complutense Madrid, Spain.

**Running Title:** NLRP3 inflammasome and cardiac aging

**Corresponding Authors:**

Dr. Mario D. Cordero

Institute of Nutrition and Food Technology "José Mataix Verdú", Department of Physiology, Biomedical Research Center, University of Granada, 18100 Granada, Spain. +34-958-241-000 (ext. 20316), Email: [mdcormor@ugr.es](mailto:mdcormor@ugr.es)

**Keywords:** NLRP3-inflammasome, cardiac aging, autophagy, longevity

**SUPPLEMENTARY INFORMATION**

**Table S1:** Biochemical parameters in plasma of NLRP3 -/- and *wild-type* mice during aging.

|  | WT | | NLRP3 -/- | | |
| --- | --- | --- | --- | --- | --- |
| Parameters | Young | Old | | Young | Old |
| Cholesterol (mg/dL) | 300.1±15.4 | 351.3±12.4** | | 308.1±11.6 | 273.1±18.7 ^aaa^ |
| Triglycerides (mg/dL) | 35.4±6.9 | 25.9±7.1 | | 44.2±8.3 | 31.5±12.4 |
| Glucose (mg/dL) | 125.8±5.5 | 216.4±10.8*** | | 144.5±8.6 | 116.1±18.8 ^aaa^ |
| IGF-1 (ng/mL) | 52.1±3.3 | 61.4±3.11 | | 34.1±4.6^aaa^ | 37.8±6.4^aaa^ |
| Albumin (mg/dL) | 2.39±0.24 | 2.23±0.15 | | 2.64±0.11 | 2.82±0.12 |
| Bilirubin (mg/dL) | 0.17±0.03 | 0.15±0.02 | | 0.14±0.03 | 0.12±0.02 |
| Lactate dehydrogenase (UL) | 1398 (155) | 2210 (325)*** | | 1173 (197) | 1411 (351) ^aaa^ |
| Ala aminotransferase (UL) | 321.5±88 | 551.83±95*** | | 339.21±89 | 352.1±112 ^aaa^ |
| Asp aminotransferase (UL) | 328.7±79 | 478.3±85*** | | 341.55± 112 | 369.1±89 ^aaa^ |
| Creatine phosphokinase (UL) | 3759±956 | 6115±750*** | | 3271±742 | 3412±539 ^aaa^ |
| Uric Acid (µMol/dL) | 25.24±2.2 | 31.29±1.7** | | 29.05±2.1 | 28.11±1.7 |
| Creatinine (mg/dL) | 0.59±0.03 | 1.53±0.41*** | | 0.90±0.15 | 1.04±0.13 ^a^ |
| TNF-α (pg/mL) | 7.9±1.3 | 35.8±3.6*** | | 6.8±1.9 | 30.9±4.2*** |
| IL-6 (pg/mL) | 21.3±4.1 | 89.5±6.8*** | | 16.9±2.3 | 78.2±5.6*** |
| IL-8 (pg/mL) | 15.8±5.7 | 71.5±7.3*** | | 10.1±1.9 | 66.8±6.3*** |

Values are presented as mean ± SEM. UL, units per litre. * *P˂0.05, **P˂0.005, ***P˂0.001* young versus old; *^aaa^P˂0.001 versus* WT. (n=10).


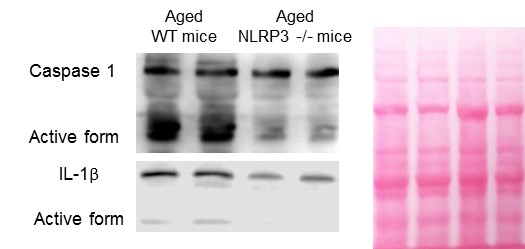


**Supplementary Figure 1.** (A) Western blot analysis showing reduced levels in the Active forms of Caspase 1 and IL-1β in the heart of aged NLRP3 -/- mice compared with aged WT.

**Table S2.** Individual values of heart weight to body weight ratios of 15 representative mice per condition.

|  | **WT Young** | **WT Old** | **NLRP3 -/- Young** | **NLRP3 -/- Old** |
| --- | --- | --- | --- | --- |

| **1** | 0,00544656 | 0,00595654 | 0,0053586 | 0,00547115 |
| --- | --- | --- | --- | --- |
| **2** | 0,00532842 | 0,00590824 | 0,00559116 | 0,0056549 |
| **3** | 0,00553299 | 0,00622328 | 0,00531664 | 0,00600908 |
| **4** | 0,00539219 | 0,00617978 | 0,00584407 | 0,0056636 |
| **5** | 0,00540007 | 0,00625723 | 0,00555598 | 0,00589816 |
| **6** | 0,00554221 | 0,00646595 | 0,00542785 | 0,00612802 |
| **7** | 0,00536042 | 0,0061719 | 0,0057432 | 0,00599136 |
| **8** | 0,0053317 | 0,00637173 | 0,00568227 | 0,00590793 |
| **9** | 0,00513985 | 0,00604161 | 0,00573671 | 0,00593507 |
| **10** | 0,00534063 | 0,00657189 | 0,00545153 | 0,00595156 |
| **11** | 0,00530032 | 0,00634751 | 0,00513968 | 0,00586804 |
| **12** | 0,00529866 | 0,00616351 | 0,00536951 | 0,00586247 |
| **13** | 0,0052454 | 0,00625067 | 0,00545286 | 0,00586289 |
| **14** | 0,00536887 | 0,00624866 | 0,0054883 | 0,00592428 |
| **15** | 0,0053125 | 0,00658995 | 0,00535364 | 0,0058491 |


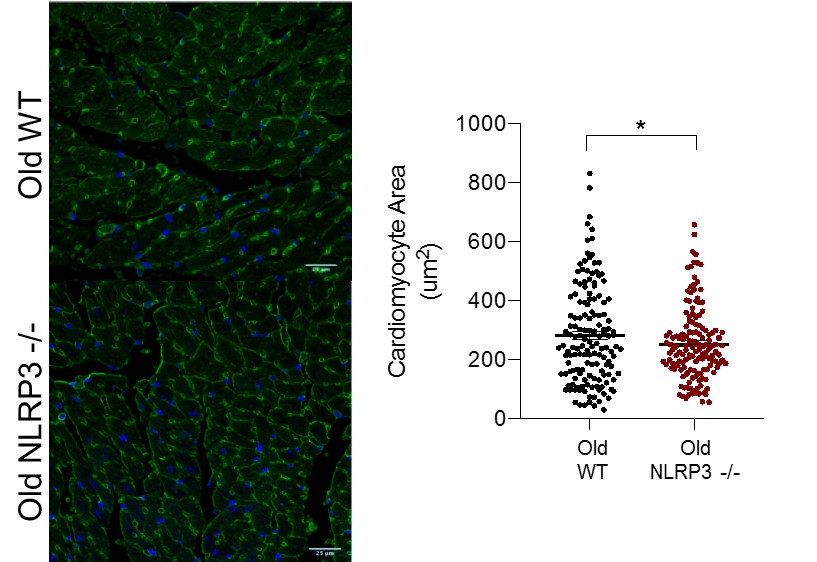


**Supplementary Figure 2.** Wheat Germ Agglutinin Staining in the heart of aged WT and NLRP3 -/- mice and quantification (4 animals per genotype). All data are presented as means ± SEM; *P < 0.05.


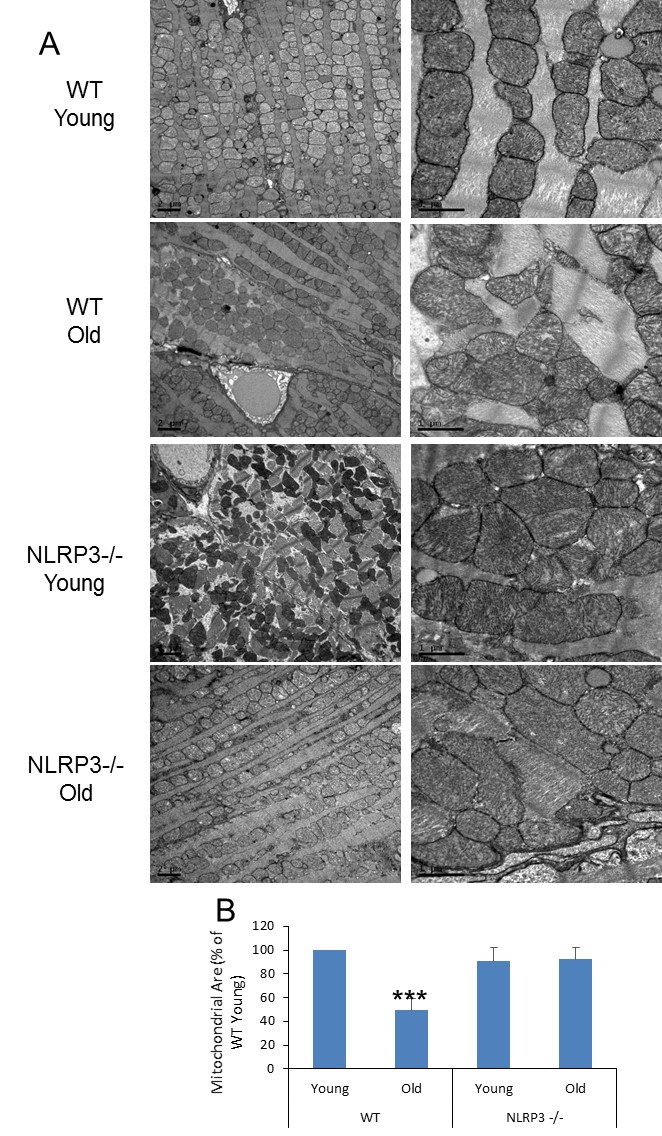


**Supplementary Figure 3.** (A) Representative TEM images of mouse left ventricle from Young and old WT and NLRP3 -/- mice (3 animals per genotype). (B) Morphometric analysis of mitochondrial area. All data are presented as means ± SEM, n = 10 mice; ***P < 0.001.


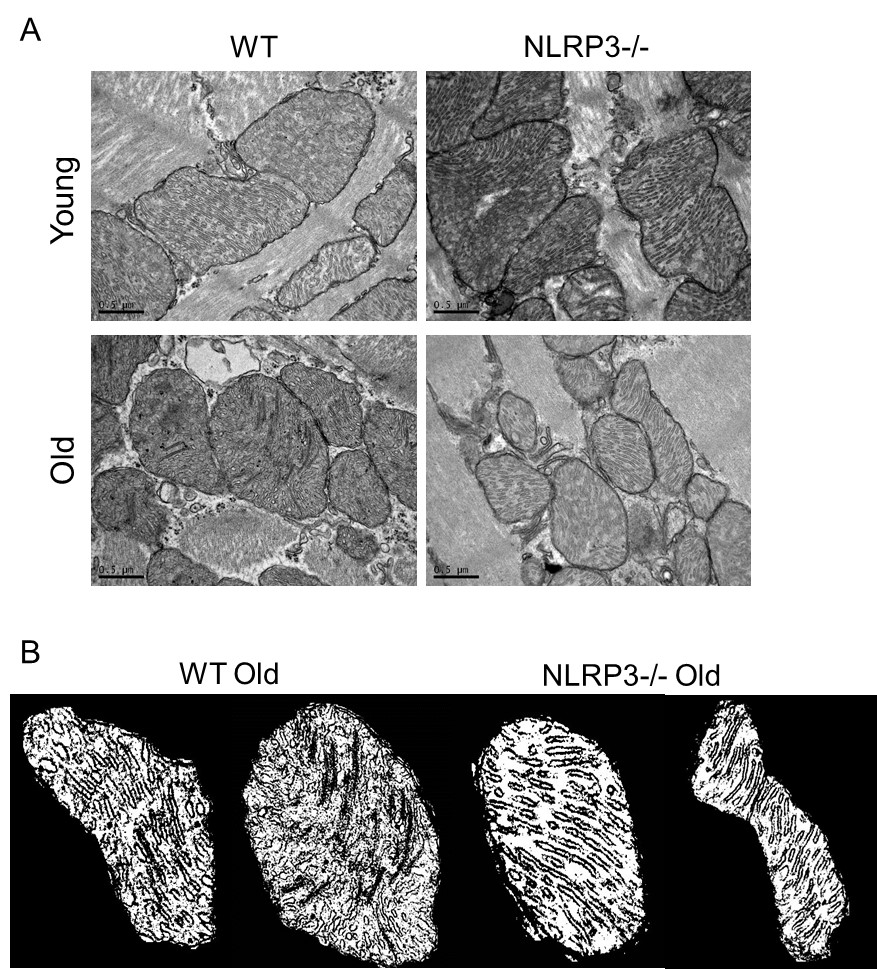


**Supplementary Figure 4.** (A) Representative TEM images of mitochondria from Young and old WT and NLRP3 -/- mice (3 animals per genotype). (B) Representative masks of individual heart mitochondria from old WT and NLRP3 -/- mice.


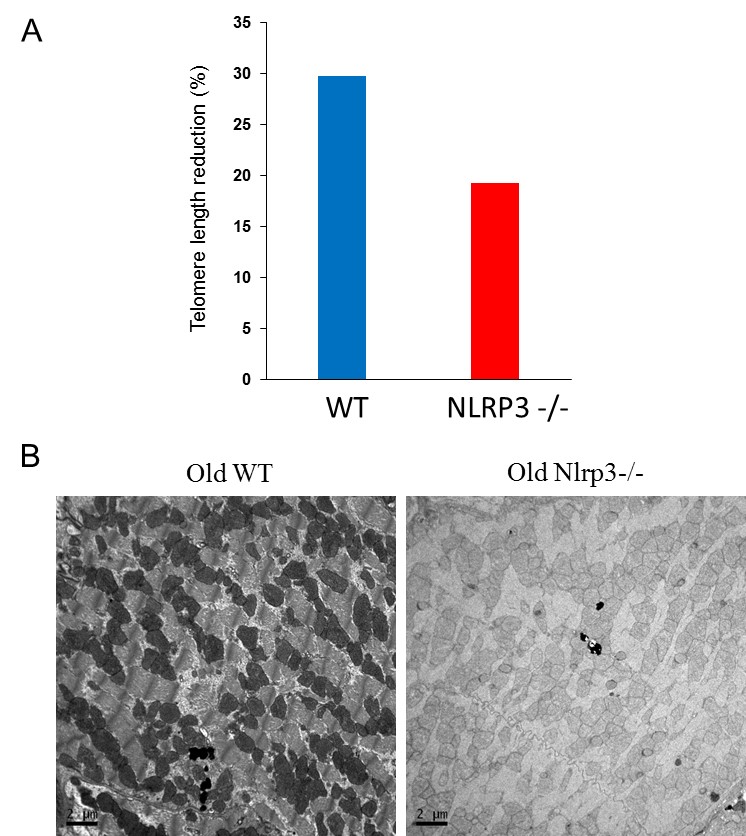


**Supplementary Figure 5.** (A) Telomere length reduction in old mice during the second year of life in WT mice. (B) Representative heart TEM images illustrating differences in lipofuscin.


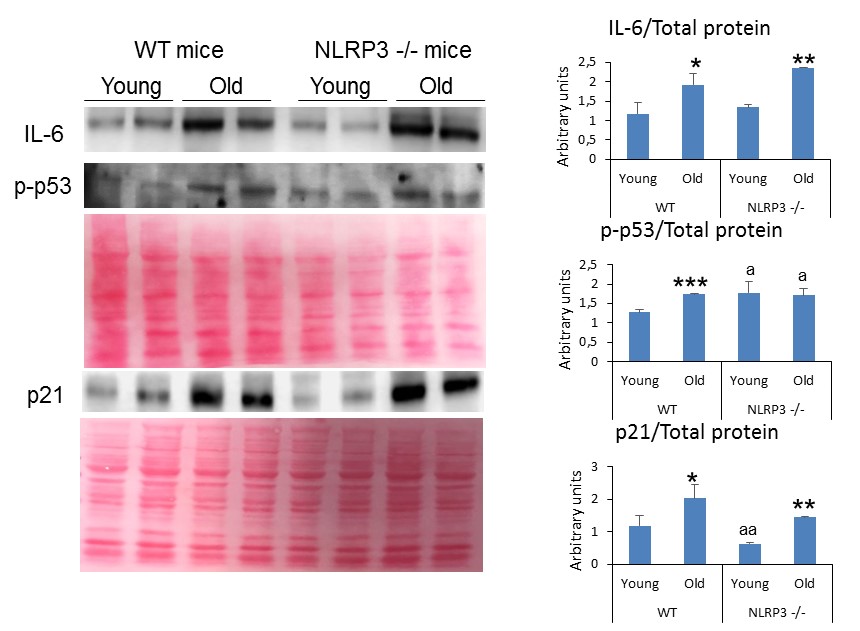


**Supplementary Figure 6.** Western blot analysis showing the protein expression of senescence markers IL-6, p21 ad p-p53 in the heart of Young and aged NLRP3 -/- mice compared with aged WT. All data are presented as means ± SEM, n = 4 mice; *P < 0.05, **P < 0.005, ***P < 0.001 young *vs* old mice. ^a^P < 0.05, ^aa^P < 0.005, WT *vs* NLRP3 -/- mice.


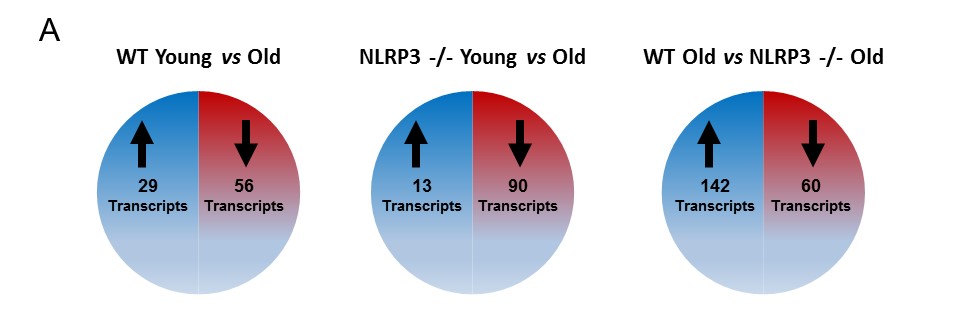


**Supplementary Figure 7**. A Diagram showing the number of genes up-and down-regulated in young and old WT and NLRP3 -/-.

**
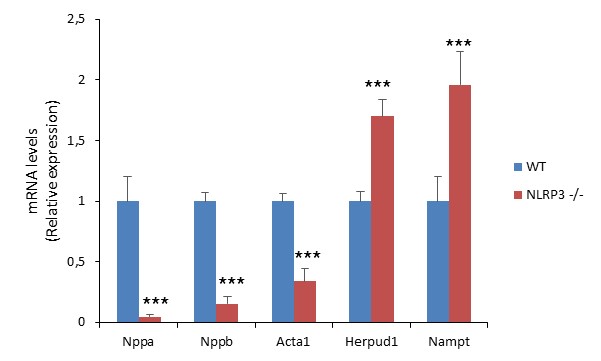
**

**Supplementary Figure 8.** mRNA expression analysis in heart by quantitative real-time PCR. Relative expression values were normalized to those of vehicle mice. All data are presented as means ± SEM, n =3 mice per group.


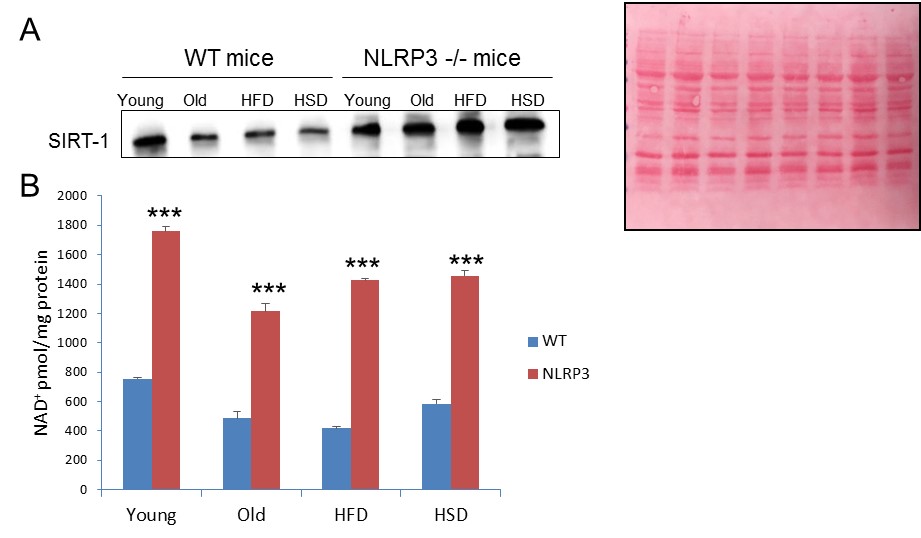


**Supplementary Figure 9.** SIRT1 protein expression (A) and NAD+ content (B) in cardiac tissues from Young and old WT and NLRP3 -/- mice and fed with high fat diet (HFD) and high sugar diet (HSD). All data are presented as means ± SEM, n = 10 mice; ***P < 0.001.

**Table S3.** **Microarray data.** Genes significantly down-regulated with aging (FDR<0.05, more than 2 fold).

| **Gene Symbol** | **Fold Change (linear) (wt Old vs. NLRP3-/- Old)** | **ANOVA p-value (wt Old vs. NLRP3-/- Old)** | **FDR p-value (wt Old vs. NLRP3-/- Old)** | **Gene Accession** |
| --- | --- | --- | --- | --- |
| Nampt | -3,23 | 0,000104 | 0,725489 | NM_021524 |
| Gpr22 | -3,16 | 0,004631 | 0,775506 | NM_175191 |
| Dbp | -3,03 | 0,042778 | 0,775506 | NM_016974 |
| Herpud1 | -2,85 | 0,003404 | 0,775506 | NM_022331 |
| Mlf1 | -2,4 | 0,068472 | 0,775506 | NM_001039543 |
| Dhrs4 | -2,39 | 0,055669 | 0,775506 | NM_001037938 |
| Kiz | -2,17 | 0,050594 | 0,775506 | NM_001033298 |
| Wee1 | -2,11 | 0,005452 | 0,775506 | NM_009516 |

**Table S4.** **Microarray data.** Genes significantly up-regulated with aging (FDR<0.05, more than 2 fold).

| **Gene Symbol** | **Fold Change (linear) (wt Old vs. NLRP3-/- Old)** | **ANOVA p-value (wt Old vs. NLRP3-/- Old)** | **FDR p-value (wt Old vs. NLRP3-/- Old)** | **Gene Accession** |
| --- | --- | --- | --- | --- |
| Nppa | 418,26 | 0,038899 | 0,775506 | NM_008725 |
| Acta1 | 35,69 | 0,03478 | 0,775506 | NM_001272041 |
| Kdr | 9,35 | 0,056718 | 0,775506 | NM_010612 |
| Rrad | 8,34 | 0,028376 | 0,775506 | NM_019662 |
| Rcan1 | 8,05 | 0,000741 | 0,725489 | NM_001081549 |
| Thbs1 | 7,63 | 0,013941 | 0,775506 | NM_011580 |
| Serpina3n | 7,59 | 0,031951 | 0,775506 | NM_009252 |
| Nppb | 6,58 | 0,031957 | 0,775506 | NM_001287348 |
| Rnu6 | 5,91 | 0,041046 | 0,775506 | NR_003027 |
| Cdkn1a | 5,7 | 0,00024 | 0,725489 | NM_001111099 |
| Mt2 | 5,49 | 0,005336 | 0,775506 | NM_008630 |
| Clic5 | 5,43 | 0,029888 | 0,775506 | NM_172621 |
| Slc41a3 | 5,4 | 0,000379 | 0,725489 | NM_001037493 |
| Pgam1-ps2 | 5,22 | 0,026011 | 0,775506 | ENSMUST00000091336 |
| Tnfrsf12a | 5,17 | 0,010429 | 0,775506 | NM_001161746 |
| Adgrg1 | 4,98 | 0,052936 | 0,775506 | NM_00119889 |
| Trim72 | 4,93 | 0,033887 | 0,775506 | NM_001079932 |
| Eif3a | 4,79 | 0,068421 | 0,775506 | NM_010123 |
| Tbx20 | 4,77 | 0,045391 | 0,775506 | NM_001205085 |
| Ppp1r3c | 4,69 | 0,00285 | 0,775506 | NM_016854 |
| Jph2 | 4,66 | 0,060337 | 0,775506 | NM_001205076 |
| Ktn1 | 4,63 | 0,06524 | 0,775506 | NM_008477 |
| Clock | 4,57 | 0,011749 | 0,775506 | NM_001289826 |
| Mir467c | 4,54 | 0,002001 | 0,775506 | NR_030571 |
| Ankrd23 | 4,46 | 0,014217 | 0,775506 | NM_153502 |
| Ptpn11 | 4,11 | 0,054054 | 0,775506 | NM_001109992 |
| Epha4 | 4,03 | 0,061651 | 0,775506 | NM_007936 |
| Cltc | 3,99 | 0,03151 | 0,775506 | NM_001003908 |
| Parm1 | 3,95 | 0,037135 | 0,775506 | NM_145562 |
| Fuca2 | 3,76 | 0,00424 | 0,775506 | NM_025799BC094449 |
| 2810474O19Rik | 3,76 | 0,006324 | 0,775506 | NM_001289661 |
| Lpin1 | 3,68 | 0,028853 | 0,775506 | NM_001130412 |
| Fbn1 | 3,67 | 0,021784 | 0,775506 | NM_007993 |
| Slc38a2 | 3,66 | 0,05876 | 0,775506 | NM_175121 |
| Plxna2 | 3,64 | 0,046632 | 0,775506 | NM_008882 |
| Uck2 | 3,55 | 0,007241 | 0,775506 | ENSMUST00000053686 |
| Acta2 | 3,53 | 0,030275 | 0,775506 | NM_007392 |
| Ednra | 3,42 | 0,067714 | 0,775506 | NM_010332 |
| Hspa5 | 3,38 | 0,027612 | 0,775506 | NM_001163434 |
| Plxnd1 | 3,36 | 0,012069 | 0,775506 | NM_026376 |
| Vegfa | 3,36 | 0,063511 | 0,775506 | NM_001025250 |
| Larp1 | 3,29 | 0,06288 | 0,775506 | NM_028451 |
| Prob1 | 3,2 | 0,049 | 0,775506 | NM_001270646 |
| Nr1d1 | 3,15 | 0,031812 | 0,775506 | NM_145434 |
| Hsp90aa1 | 3,07 | 0,002804 | 0,775506 | NM_010480 |
| Ctsl | 3 | 0,016962 | 0,775506 | NM_009984 |
| Hsph1 | 2,99 | 0,005889 | 0,775506 | NM_013559 |
| Cd93 | 2,99 | 0,042868 | 0,775506 | NM_010740 |
| Cyp1b1 | 2,93 | 0,041465 | 0,775506 | NM_009994 |
| Plin3 | 2,91 | 0,00168 | 0,775506 | NM_025836 |
| Akap1 | 2,88 | 0,052964 | 0,775506 | NM_001042541 |
| Rel | 2,87 | 0,044881 | 0,775506 | NM_009044 |
| Kremen1 | 2,87 | 0,056047 | 0,775506 | NM_032396 |
| P4ha1 | 2,81 | 0,002965 | 0,775506 | NM_011030 |
| Tgm2 | 2,78 | 0,005924 | 0,775506 | NM_009373 |
| Lrrc8c | 2,75 | 0,008367 | 0,775506 | NM_133897 |
| Plod1 | 2,75 | 0,028084 | 0,775506 | NM_011122 |
| Arntl | 2,73 | 0,000075 | 0,725489 | NM_001243048 |
| Plpp3 | 2,69 | 0,027728 | 0,775506 | NM_080555 |
| Ide | 2,69 | 0,037083 | 0,775506 | NM_031156 |
| Col4a2 | 2,69 | 0,038395 | 0,775506 | NM_009932 |
| Zfp568 | 2,69 | 0,042048 | 0,775506 | NM_001033355 |
| Ntn1 | 2,67 | 0,061582 | 0,775506 | NM_008744 |
| Ttc17 | 2,66 | 0,069429 | 0,775506 | NM_183106 |
| Myot | 2,63 | 0,024762 | 0,775506 | NM_001033621 |
| Pam | 2,62 | 0,027936 | 0,775506 | NM_013626 |
| Osmr | 2,61 | 0,011959 | 0,775506 | NM_011019 |
| Gns | 2,61 | 0,045234 | 0,775506 | NM_029364 |
| Enah | 2,61 | 0,048235 | 0,775506 | NM_001083120 |
| Dmxl2 | 2,6 | 0,042827 | 0,775506 | NM_172771 |
| Sparc | 2,58 | 0,012543 | 0,775506 | NM_009242 |
| Irs1 | 2,57 | 0,029511 | 0,775506 | NM_010570 |
| Srl | 2,57 | 0,044193 | 0,775506 | NM_175347 |
| Adprhl1 | 2,56 | 0,035527 | 0,775506 | NM_172750 |
| Itga5 | 2,54 | 0,069865 | 0,775506 | NM_010577 |
| Zdhhc5 | 2,53 | 0,022932 | 0,775506 | NM_144887 |
| Trak2 | 2,53 | 0,06193 | 0,775506 | NM_172406 |
| Sacm1l | 2,5 | 0,010196 | 0,775506 | NM_030692 |
| Mical2 | 2,5 | 0,012148 | 0,775506 | NM_001193305 |
| Optn | 2,49 | 0,01166 | 0,775506 | NM_181848 |
| Adipor2 | 2,48 | 0,039892 | 0,775506 | NM_197985 |
| Gda | 2,46 | 0,042532 | 0,775506 | NM_010266 |
| Gbp10; Gbp6 | 2,45 | 0,002531 | 0,775506 | NM_001039646 |
| Itga1; Pelo | 2,45 | 0,055169 | 0,775506 | NM_001033228 |
| Prkar1a | 2,42 | 0,000971 | 0,775506 | NM_021880 |
| Mlec | 2,4 | 0,053743 | 0,775506 | NM_175403 |
| Hsp90b1 | 2,35 | 0,05891 | 0,775506 | NM_011631 |
| Alas1 | 2,34 | 0,013525 | 0,775506 | NM_020559 |
| Hif1an | 2,33 | 0,055884 | 0,775506 | NM_176958 |
| Nr4a3 | 2,32 | 0,011718 | 0,775506 | NM_015743 |
| Twf2 | 2,3 | 0,001343 | 0,775506 | NM_011876 |
| Stat3 | 2,3 | 0,003969 | 0,775506 | NM_011486 |
| Murc | 2,3 | 0,016755 | 0,775506 | NM_026509 |
| Itga6 | 2,27 | 0,068226 | 0,775506 | NM_001277970 |
| Clu | 2,26 | 0,033104 | 0,775506 | NM_013492 |
| Rab11fip5; Mir705 | 2,24 | 0,009407 | 0,775506 | NM_001003955 |
| B4galt6 | 2,24 | 0,015324 | 0,775506 | NM_019737 |
| Mcam | 2,23 | 0,015353 | 0,775506 | NM_023061 |
| Dcaf7 | 2,23 | 0,066398 | 0,775506 | NM_027946 |
| Prps2 | 2,23 | 0,068735 | 0,775506 | NM_026662 |
| Uck2 | 2,22 | 0,043249 | 0,775506 | NM_030724 |
| Nacc1 | 2,21 | 0,017817 | 0,775506 | NM_025788 |
| Ap1b1 | 2,21 | 0,02155 | 0,775506 | NM_001243043 |
| Col4a1 | 2,21 | 0,023836 | 0,775506 | NM_009931 |
| Rbpms2 | 2,19 | 0,046395 | 0,775506 | NM_028030 |
| Elovl5 | 2,19 | 0,049429 | 0,775506 | NM_134255 |
| Arfgef2 | 2,19 | 0,053697 | 0,775506 | NM_001085495 |
| Csf1 | 2,18 | 0,030025 | 0,775506 | NM_001113529 |
| Hspa4 | 2,15 | 0,026764 | 0,775506 | NM_008300 |
| Sulf2 | 2,15 | 0,064398 | 0,775506 | NM_001252578 |
| Rrp12 | 2,14 | 0,028827 | 0,775506 | NM_199447 |
| Perm1 | 2,14 | 0,03265 | 0,775506 | NM_172417 |
| Tigar | 2,12 | 0,009492 | 0,775506 | NM_177003 |
| Tubb2a | 2,12 | 0,014865 | 0,775506 | NM_009450 |
| sep-11 | 2,12 | 0,056453 | 0,775506 | NM_001009818 |
| Flnb | 2,12 | 0,061557 | 0,775506 | NM_134080 |
| Slc35f6; Mir5625 | 2,12 | 0,062831 | 0,775506 | NM_175675 |
| Wdr1 | 2,11 | 0,004702 | 0,775506 | NM_011715 |
| Trp53i11 | 2,11 | 0,008959 | 0,775506 | NM_001025246 |
| Pds5a | 2,11 | 0,031806 | 0,775506 | NM_001081321 |
| Hif1a | 2,11 | 0,057054 | 0,775506 | NM_010431 |
| Ganab | 2,11 | 0,057258 | 0,775506 | NM_008060 |
| Pfkp | 2,1 | 0,001333 | 0,775506 | NM_019703 |
| Chordc1 | 2,1 | 0,004947 | 0,775506 | NM_025844 |
| Sox18 | 2,1 | 0,00755 | 0,775506 | NM_009236 |
| Atp6v0a2 | 2,1 | 0,012376 | 0,775506 | NM_011596 |
| Cpxm2 | 2,1 | 0,02969 | 0,775506 | NM_018867 |
| Azin1 | 2,09 | 0,005213 | 0,775506 | NM_001102458 |
| Pfkfb2; C4bp-ps1 | 2,09 | 0,019886 | 0,775506 | NM_001162415 |
| Lrp5 | 2,09 | 0,029533 | 0,775506 | NM_008513 |
| Sec31a | 2,09 | 0,04515 | 0,775506 | NM_026969 |
| Tnks2 | 2,09 | 0,055745 | 0,775506 | NM_001163635 |
| Spast | 2,08 | 0,05102 | 0,775506 | NM_001162870 |
| Myh9 | 2,08 | 0,06249 | 0,775506 | NM_022410 |
| Tmlhe | 2,07 | 0,035497 | 0,775506 | NM_138758 |
| Sf3b3 | 2,05 | 0,016504 | 0,775506 | NM_133953 |
| Rapgef1 | 2,04 | 0,028561 | 0,775506 | NM_001039086 |
| Srf | 2,04 | 0,033731 | 0,775506 | NM_020493 |
| Baiap2l1 | 2,03 | 0,01153 | 0,775506 | NM_025833 |
| Sema7a | 2,03 | 0,023908 | 0,775506 | NM_011352 |
| Ap3d1 | 2,02 | 0,022434 | 0,775506 | NM_007460 |
| Cyp2u1 | 2,01 | 0,038943 | 0,775506 | NM_027816 |
| Parvb | 2,01 | 0,060061 | 0,775506 | NM_133167 |
| Art3 | 2,01 | 0,066082 | 0,775506 | NM_181728 |
| Il4ra | 2 | 0,000499 | 0,725489 | NM_001008700 |
| Tspan9 | 2 | 0,007208 | 0,775506 | NM_175414 |
| Dctn4 | 2 | 0,047573 | 0,775506 | NM_026302 |

**Table S4**: Primers of the gene expression study.

| **Gen amplificado** | **Secuencia** |
| --- | --- |
| Nppa | Primer forward: CCTAAGCCCTTGTGGTGTGT  Primer reverse: CAGAGTGGGAGAGGCAAGAC |
| Nppb | Primer forward: CAGCTCTTGAAGGACCAAGG  Primer reverse: AGACCCAGGCAGAGTCAGAA |
| Acta1 | Primer forward: GCATGCAGAAGGAGATCACA  Primer reverse: TTGTCGATTGTCGTCCTGAG |
| Thbs1 | Primer forward: CCAAAGCCTGCAAGAAAGAC  Primer reverse: CCTGCTTGTTGCAAACTTGA |
| Nampt | Primer forward: AGGGGTTAGCAGGTGATCCT  Primer reverse: TAGTGCGGGCTAGCCTAAAA |
| Herpud1 | Primer forward: ACAAAGGGTGCTGAATCCAC  Primer reverse: CCTTGGAAAGTCTGCTGGAC |
| β-actin | Primer forward: TGTTACCAACTGGGACGACA  Primer reverse: GGGGTGTTGAAGGTCTCAAA |

Original Western blots


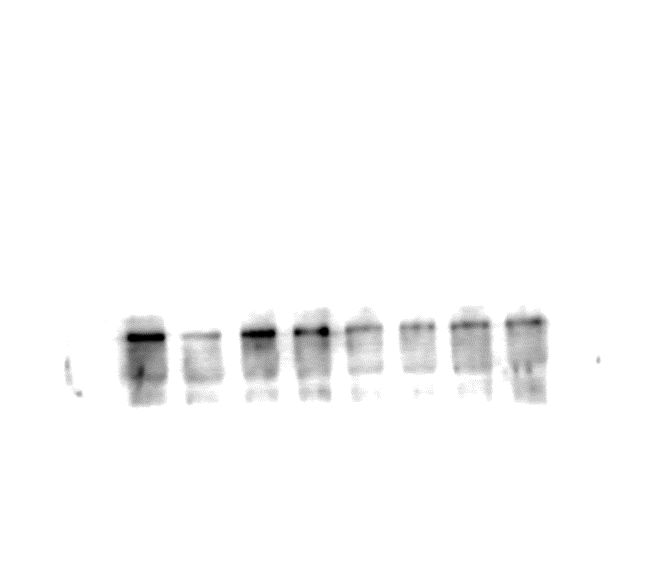

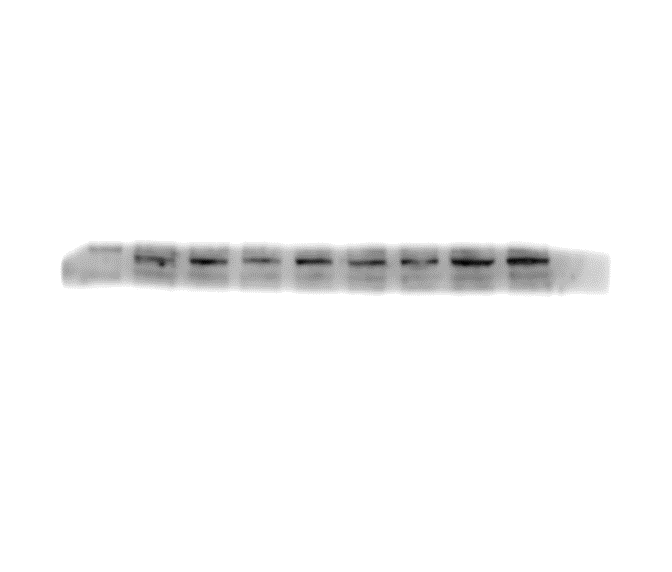

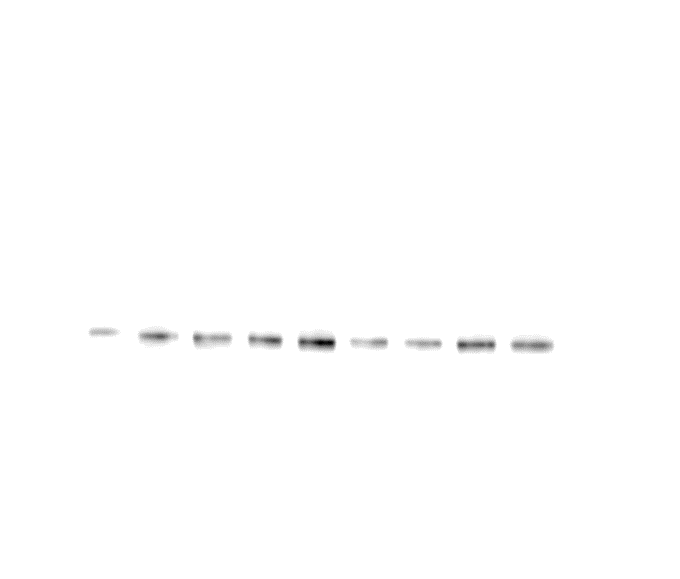

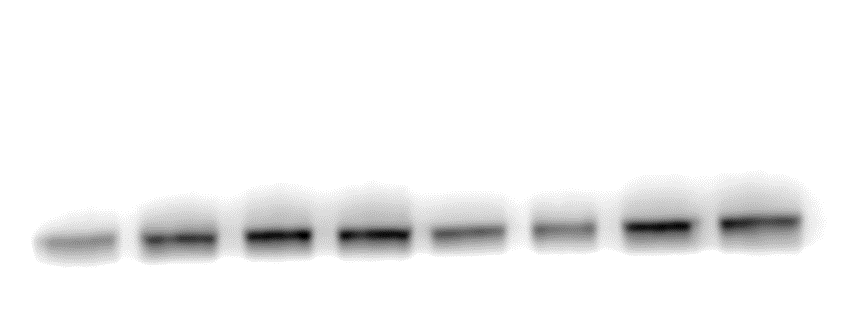

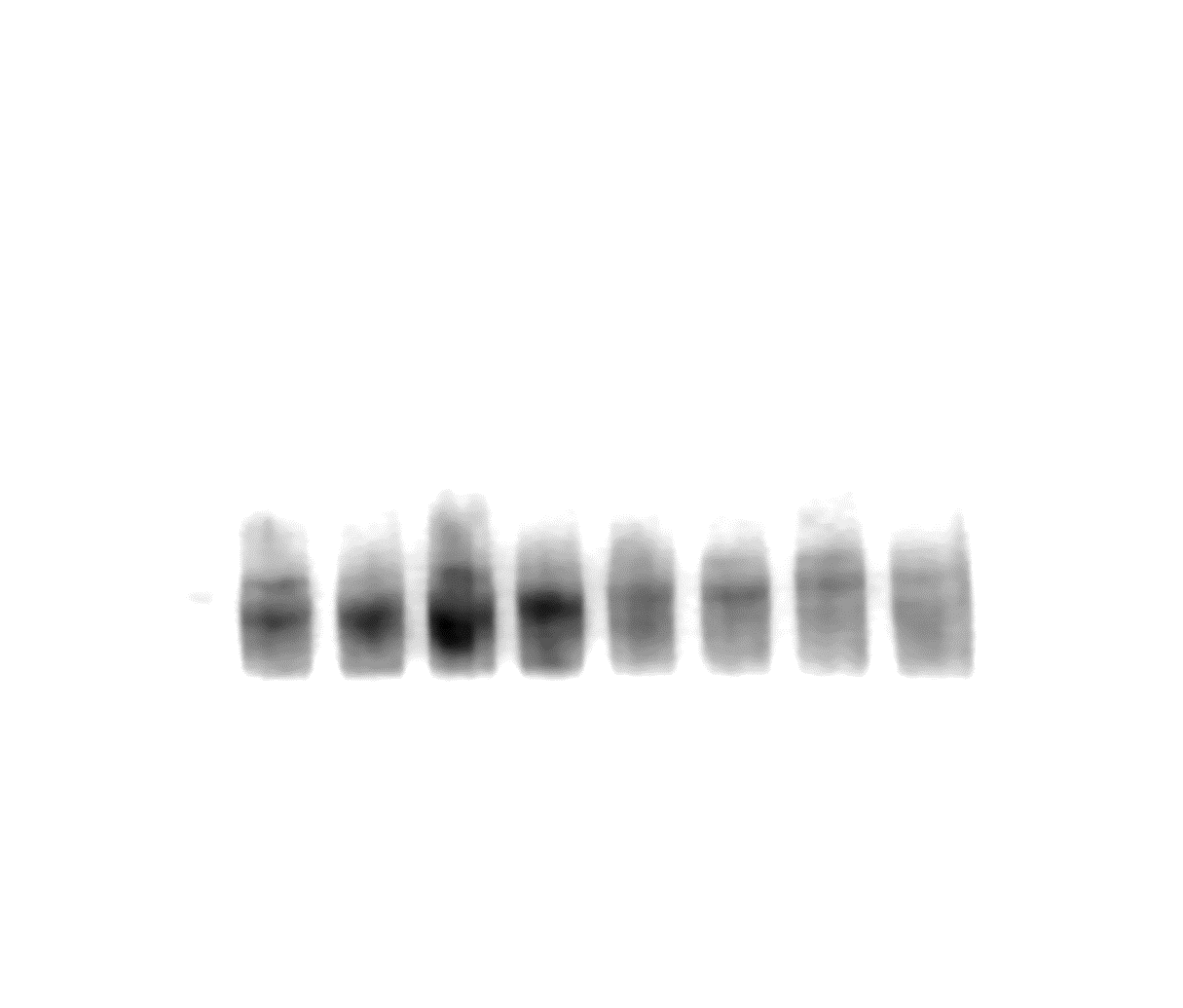

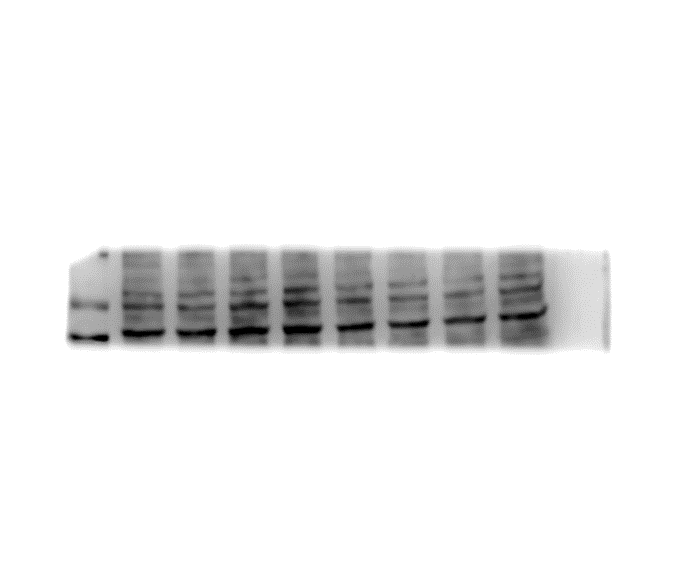


P62

ATG12

p-PI3K

PI3K

p-mTOR

mTOR


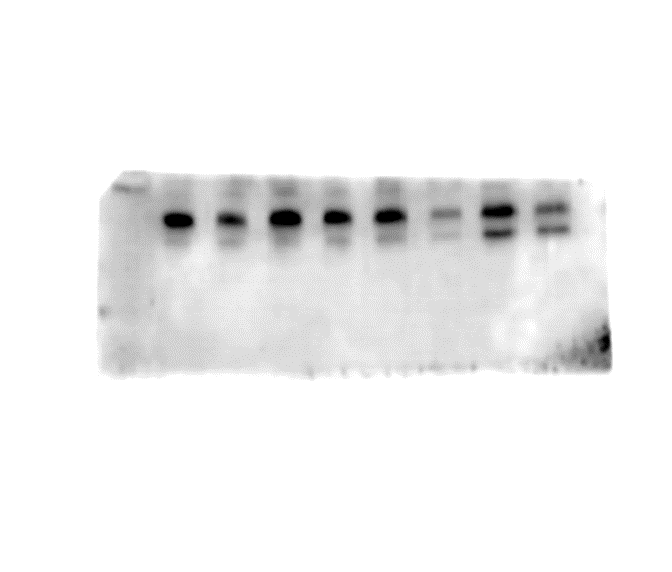

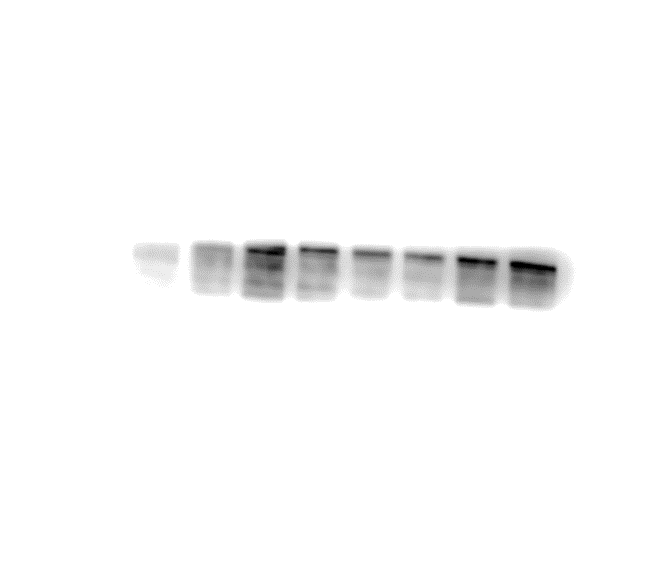


LC3

Beclin1
